# Surface Treatment of Dental Mini-Sized Implants and Screws: A Systematic Review with Meta-Analysis

**DOI:** 10.3390/jfb15030068

**Published:** 2024-03-10

**Authors:** Ana Luísa Figueiredo, Raquel Travassos, Catarina Nunes, Madalena Prata Ribeiro, Mariana Santos, Flavia Iaculli, Anabela Baptista Paula, Carlos Miguel Marto, Francisco Caramelo, Inês Francisco, Francisco Vale

**Affiliations:** 1 Institute of Orthodontics, Faculty of Medicine, University of Coimbra, 3000-354 Coimbra, Portugal; uc2018277869@student.uc.pt (A.L.F.); uc47231@uc.pt (R.T.); uc47230@uc.pt (C.N.); uc2020181154@student.uc.pt (M.P.R.); uc2014107642@student.uc.pt (M.S.); fvale@fmed.uc.pt (F.V.); 2 Laboratory for Evidence-Based Sciences and Precision Dentistry (LACBE–MDP), Faculty of Medicine, University of Coimbra, 3000-354 Coimbra, Portugal; cmiguel.marto@uc.pt (C.M.M.); fcaramelo@fmed.uc.pt (F.C.); 3 Institute for Clinical and Biomedical Research (iCBR), Area of Environment Genetics and Oncobiology (CIMAGO), Faculty of Medicine, University of Coimbra, 3000-354 Coimbra, Portugal; 4 Clinical Academic Center of Coimbra (CACC), 3000-354 Coimbra, Portugal; 5 Department of Neurosciences, Reproductive and Odontostomatological Sciences, University of Naples “Federico II”, 80138 Naples, Italy; flavia.iaculli@unina.it; 6 Institute of Integrated Clinical Practice, Faculty of Medicine, University of Coimbra, 3000-354 Coimbra, Portugal; 7 Institute of Experimental Pathology, Faculty of Medicine, University of Coimbra, 3000-354 Coimbra, Portugal

**Keywords:** miniscrews, skeletal anchorage, stability, surface properties, orthodontics

## Abstract

Miniscrews are devices that allow for absolute skeletal anchorage. However, their use has a higher failure rate (10–30%) than dental implants (10%). To overcome these flaws, chemical and/or mechanical treatment of the surface of miniscrews has been suggested. There is no consensus in the current literature about which of these methods is the gold standard; thus, our objective was to carry out a systematic review and meta-analysis of the literature on surface treatments of miniscrews. The review protocol was registered (PROSPERO CRD42023408011) and is in accordance with the PRISMA guidelines. A bibliographic search was carried out on PubMed via MEDLINE, Cochrane Library, Embase and Web of Science. The initial search of the databases yielded 1684 results, with 98 studies included in the review, with one article originating from the search in the bibliographic references of the included studies. The results of this systematic review show that the protocols of miniscrew surface treatments, such as acid-etching; sandblasting, large-grit and acid-etching; photofunctionalization with ultraviolet light; and photobiomodulation, can increase stability and the success of orthodontic treatment. The meta-analysis revealed that the treatment with the highest removal torque is SLA, followed by acid-etching. On the other hand, techniques such as oxidative anodization, anodization with pre-calcification and heat treatment, as well as deposition of chemical compounds, require further investigation to confirm their effectiveness.

## 1. Introduction

With the increasing use of dental implants to replace missing teeth, implants of different sizes have been manufactured to meet different clinical situations [1]. Unlike dental implants which are made up of two parts (the implant and the abutment), mini dental implants have a single-piece titanium screw with a spherical head to stabilize the prosthesis or a square prosthetic head for fixed applications instead of the classic abutment. Mini implants are smaller in diameter and are often used to stabilize dentures or as temporary anchorage devices in orthodontic treatment. They are typically around 1.0 to 2.0 mm in diameter and 6 to 10 mm in length, whereas conventional implants used in dentistry for replacing missing teeth are larger, usually ranging from 3.0 to 6.0 mm in diameter and 7 to 16 mm in length [2,3]. Orthodontic mini implants are solely used as temporary anchorage devices (TADs), providing absolute anchorage and aid in eliminating unwanted forces and deleterious tooth movements [4,5]. These devices were first described in 1997 by Kanomi [5]. The use of these systems makes it possible to perform movements that would otherwise be unachievable [6]. The use of miniscrews can be beneficial in a wide variety of orthodontic movements, such as retraction and intrusion of anterior and posterior teeth, mesialization or distalization of molars and elimination of undesirable spaces [7]. In turn, these movements will allow for the correction of deep bites, midline deviations and sagital discrepances and will help improve the Spee curve [4]. The use of TADs will ideally result in greater treatment efficacy due to the optimization of orthodontic movement. These particularities, together with their ease of use, the lack of the need for extensive surgery and an agreeable cost–benefit ratio, as well as their small size, are factors that contribute to therapeutic success and satisfaction for both patients and providers [4,5].

The stability of miniscrews is divided into two stages: primary and secondary. Primary stability is achieved by the mechanical retention of the device to the bone, and it varies according to the type of implant, mechanical characteristics, implantation condition and properties of the target bone. In the weeks following insertion, the stability of miniscrews varies according to the type of miniscrew, implantation method and properties of the target bone. In order to achieve this, it is vital to consider the characteristics of the bone, for example whether medullar or alveolar bone is present, the attributes of the implant surface and the timings of bone cell remodeling [5]. Similarly, an important characteristic of miniscrews which can influence their success is the material from which they are produced. Initially, the materials recommended for the manufacture of these devices were based on stainless steel, an alloy composed mainly of iron, nickel, chromium and carbon. With the evolution of materials and search for compounds that were more biocompatible, there was a paradigm shift, and today most of them are produced from titanium, with purity levels ranging from grade I to V [4,5].

As a result of its properties and close connection to the surrounding bone, the miniscrew has a lower loss of anchorage over time compared to other anchorage methods [8]. However, when compared to the devices from which they were initially derived, conventional dental implants, their failure rate is higher. Studies have reported that 10–30 percent of miniscrews fail, a figure that is significantly higher than the 10 percent of traditional implants [6,9,10]. The initial stability is often lacking in cases of inadequate cortical bone thickness. Furthermore, the loss of miniscrew stability can be attributed to inflammation or bone remodeling [10]. There is evidence, on the other hand, that more failures of these devices are reported in the mandible, although the literature is not in complete agreement. These results may be due, for example, to the higher density of the mandibular bone, causing higher insertion torque values, as well as necrosis due to excessive bone heating during placement and lower cortical bone production around the miniscrew [10].

A miniscrew is considered lost when it is no longer able to irreversibly anchor the fixed appliance, and thus is no longer resisting the forces created by reactions, leading to its removal and consequent need for replacement [10]. In its initial phase, loss can occur due to the device unscrewing, a situation that can be prevented by improving the technique and properties of the miniscrew [6]. Its loss is not, however, a one-off occurrence. This perpetuates complications not only in soft tissue, but also in hard tissue, namely root damage to adjacent teeth, perforation of the maxillary sinus, inflammation of the soft tissues and hypertrophy of the peripheral mucosa [11]. In order to overcome the limitations described above, surface treatment techniques for orthodontic miniscrews have been suggested. In general, these strategies aim to improve the anchoring properties so that early loss of these devices, being of mechanical and/or chemical origin, can be prevented. These techniques are intended to improve the topography of the coils and their surface roughness, promoting good adhesion and cell interaction. Certain treatments may also provide decontamination of the miniscrew length [12,13].

The miniscrew can be produced with an untreated or altered surface, maintaining only the properties of the chosen titanium alloy. In order to modify the surface, there are techniques described in the literature that aim to meet the requirements explained above, such as microgrooving, sterilization, surface anodization, sandblasting and plasma ion implantation, as well as ultraviolet light treatment [14]. Table 1 describes the main advantages and disadvantages of the surface treatments most cited in the literature.

One of the most used surface treatments to date is acid-etching of the surface of miniscrews. It primarily submerges the miniscrews in an acid, such as phosphoric acid, to improve roughness and resistance to compressive forces [19,20]. Additionally, another surface treatment commonly described is sandblasting, large-grit and acid-etching (SLA). It takes advantage of the properties of an acid and tallies it to particle abrasion with aluminium oxide, for example, to better cultivate cell adhesion to the miniscrew and augment its mechanical stability [21,22]. In recent years, these techniques have been widely investigated, but there is no consensus on which method is the gold standard. The authors, therefore, set out to carry out a systematic review and meta-analysis of the literature on possible surface treatments for miniscrews, comparing their characteristics and protocols and evaluating their influence on clinical stability.

## 2. Materials and Methods

### 2.1. Protocol

The protocol for this systematic review was registered on the PROSPERO platform, having received approval with the registration number CRD42023408011. Its organization and methodology followed the PRISMA guidelines (Preferred Reporting Items for Systematic Reviews and Meta-Analysis) [23], which resulted in the following PICO (Population, Intervention, Comparison and Outcome) question: “What is the effect of surface treatment on the mechanical stability of miniscrews in patients undergoing orthodontic treatment?”.

### 2.2. Study Strategy and Selection

After obtaining the PICO question, a comprehensive literature search was performed in the following databases: PubMed (via MEDLINE), Cochrane Library (Trials), Embase and Web of Science (all databases). For each database, different variations of the same search key were used in order to respect the particularities of each platform.

In all searches, the language filter was applied to include only studies in English, Portuguese, Spanish and French. The last search carried out in all databases was performed on 19 November 2023, by two researchers, independently. Additionally, the investigation included searching ProQuest (Database, Ebooks and Technology for research), HSRProj and Onegrey, as well as a manual search of the bibliographic references of included studies. Supplementary Materials, Table S1 summarizes the search strategies used. To better manage the results obtained, the bibliographic reference tool Endnote Web (version 21, Clarivate Analytics) was used.

Subsequently, studies eligible for review and analysis were selected. From the results obtained, all duplicate studies were first removed using the Find duplicates tool on EndNote Web. After extraction, three independent reviewers scrutinized the remaining studies (A.L.F., R.T., I.F.), choosing only those in compliance with the established eligibility criteria. Firstly, the selection was carried out according to the title and abstract, and, finally, the residual studies were verified, analyzing their text in full. In case of any doubt or disagreement, a fourth reviewer was contacted and consulted (F.V.).

The primary outcome was defined as the mechanical stability of the miniscrews. However, the secondary outcomes were as follows: tooth movement, periodontal health, pain and discomfort felt by the patient, possible changes in speech and aesthetics and, finally, the analysis of the cost of the treatment.

The following inclusion criteria were defined: in-vitro studies; in-vivo studies; ex-vivo studies; randomized, non-randomized, case-control and cohort clinical studies; and studies that evaluated the stability of miniscrews as the primary outcome. On the other hand, the exclusion criteria applied were as follows: editorials or books and book chapters; studies with incomplete information; case report/clinical case series; and descriptive studies.

### 2.3. Data Extraction

For each study included, three independent investigators (A.L.F., R.T., I.F.) extracted the following information: authors, year of publication, study design (in vitro, in vivo, ex vivo or clinical), sample characteristics such as species (if applicable), sex and age, sample size, test group and control group, material and protocol (application time and dose) used for treatment, outcomes evaluated, type of intervention evaluation, main follow-up period(s), results and conclusions.

A first reviewer performed data extraction and synthesis (A.L.F.). This condensation was then reviewed and, when necessary, corrected, by two other researchers (R.T. and I.F.), with a fourth reviewer being contacted in case of doubts or disagreement (F.V.).

### 2.4. Quality Assessment

In order to assess the quality of the methodology of the studies included in this review, we took advantage of several already validated tools in order to assess the risk of bias of each of them. Two reviewers (A.L.F. and R.T.) independently analyzed the quality of the studies, with a third (I.F.) mediating any disagreements.

With regard to in vitro studies, these were evaluated using Faggion Jr.’s guidelines for reporting pre-clinical studies related to dental materials [24]. Equivalently, the SYRCLE tool (Systematic Review Center for Laboratory Animal Experimentation, Netherlands) was used when analyzing the risk of bias of in vivo studies [25]. Finally, for clinical studies, the Cochrane guidelines were used (RoB-2 and ROBINS-I tools) [26,27].

### 2.5. Statistical Analysis

The objective of this study is to assess and compare surface treatments applied to miniscrews, specifically examining SLA (sandblasting, large-grit and acid-etching) and AE (acid-etching). The evaluation criteria include removal torque (RTV), insertion torque (ITV), success rate (SR) and bone contact interface (%BIC). The analysis encompasses studies conducted in patients, along with in vivo studies, and incorporates meta-analyses of the gathered data.

In the analysis, the primary outcome measures were the raw mean and odds ratio, utilizing random-effects models for data processing. To assess the heterogeneity among the included studies, both the Q-test for heterogeneity and the I² statistic were calculated, offering insights into the variability of effect sizes across studies. Each meta-analysis was summarized through the creation of a forest plot. The statistical procedures were executed using the “metafor” package in R (version 4.4-0, Wolfgang Viechtbauer), and the plots were generated using MS^®^ Excel^®^ (version 16.16.27, Microsoft, Redmond, WA, USA). 

## 3. Results

### 3.1. Study Selection

**Figure 1:**
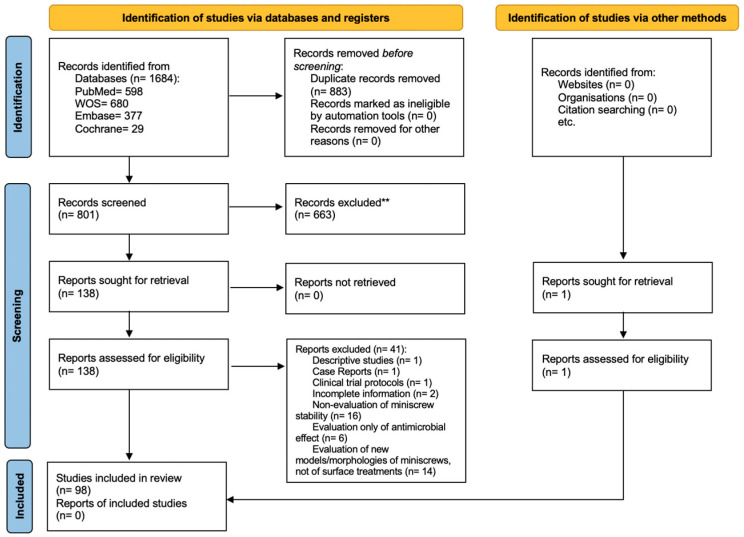
PRISMA flowchart diagram.

The initial search was conducted in various databases and resulted in 1684 studies. After the identification and removal of the 883 duplicates, the titles and abstracts were read and 633 studies were excluded. Subsequently, 138 potentially relevant references were read in full. The complete reading resulted in the exclusion of 41 additional articles. In parallel, after searching the references of the results obtained in the search in the databases, one gray literature article was included. Thus, 98 studies were included in this systematic review. The process of identification, screening and eligibility is summarized in the PRISMA flowchart (Figure 1).

### 3.2. Included Studies Characteristics

The present systematic review included 41 in vitro studies, 56 studies in vivo and 16 clinical trials (11 randomized and 5 non-randomized). The characteristics and results of the previously mentioned studies are described below.

#### 3.2.1. In Vitro Studies

The most commonly mentioned surface treatments were as follows: oxidative anodization in 8 studies; SLA (sandblasting, large-grit and acid-etching) in 5 studies; AE (acid-etching) in 6 studies; photofunctionalization in 3 studies; deposition of different fluids, solutions and chemical compounds in 11 studies; and different sterilization methods in 11 studies.

In order to assess the effect of the surface treatments, follow-up times ranged from 12 h 20 to 10 [28] and 12 weeks [29]. The in vitro studies were published between 2010 [30] and 2023 [31,32,33,34]. Table 2 presents the characteristics and results of the in vitro studies.

#### 3.2.2. In Vivo Studies

The surface treatment most used in this study design was the deposition of different fluids, solutions and chemical compounds (n = 13), followed by photofunctionalization/photobiomodulation (n = 10), SLA (n = 10), oxidative anodization (n = 10), acid-etching (n = 7) and novel auxiliary devices (n = 1).

The sample of animals included ranged from 2 [45] to 144 [67] and these studies were performed between 2003 [68] and 2023 [31,69,70,71].

Table 3 presents the characteristics and results of the in vivo studies.

#### 3.2.3. Clinical Trials

These studies were published between 2008 [16,73] and 2023 [110,111]. The number of patients included varied mostly between 20 and 40 patients, except in four studies that included 8, 9, 13 and 17 participants [14,111,112,113].

As far as randomized clinical trials are concerned, the surface treatments tested were photofunctionalization using ultraviolet light and LED (light emitting diode) in one study each and low-intensity laser photobiomodulation (LLLT) in two studies. Additionally, SLA and acid-etching were evaluated in four studies. One study researched the effects of precipitation of hydroxyapatite.

The results obtained were reported after follow-up times ranging from 3 days [114,115] to 22 months [73]. Table 4 presents the characteristics and results of the clinical trials.

### 3.3. Studied Outcomes and Chosen Tests

The majority of the included studies assessed stability as the primary outcome using various methods: measurement of insertion, removal and fracture torques; RFA (resonance frequency analysis); analysis of contact between bone and miniscrews; histological methods and electron microscopy; assessment of mobility using the Periotest; and periodontal changes. On the other hand, factors such as biocompatibility, the patient’s perception of pain (using the NRS-11 pain scale), tooth movement through comparisons between study models and, finally, the surface characteristics of the miniscrews created by surface treatment were also evaluated. In general, outcomes were assessed using experimental groups subjected to one or more surface treatments compared to an untreated control group.

### 3.4. Risk of Bias

The risk of bias analysis of the clinical trials, in vitro and in vivo studies is shown in Supplementary Materials, Tables S2–S5.

With regard to randomized clinical trials, the majority of studies had a low risk of bias, with the exception of four articles. Two studies show concerns in measuring their outcomes [114,115] and one study in deviations from the intended outcome [21]. On the other hand, one trial was classified as having a high risk of bias due to the non-referencing of the randomization method [21].

With regard to in vivo and in vitro studies, the majority of studies presented a high risk of bias. The main reasons for bias were the lack of randomization and blinding in the allocation of treatments, lack of blinding in the measurement of outcomes, failure to mention the limitations of the studies and the availability of their protocols.

### 3.5. Meta-Analysis

The analysis underwent an initial meticulous selection process in which priority was given to those articles containing indispensable information pertaining to the control and test groups. In this meta-analysis, of the 98 studies of the systematic review, 13 were included.

The emphasis was particularly on data related to the mean and standard deviation in quantitative variables such as RTV, ITV, BIC and SR. For dichotomous variables, the focus was directed towards the proportion of event occurrence in both groups, with special attention to the success rate (SR). It is noteworthy that, among the scrutinized treatments, only the SLA and AE groups exhibited a sufficient number of elements to perform a comprehensive meta-analysis. The ensuing results from the diverse meta-analyses conducted are now presented with clarity and visual coherence through forest plots.

#### 3.5.1. Clinical Studies

Regarding clinical studies, it was possible to carry out four meta-analyses.

**Figure 2:**
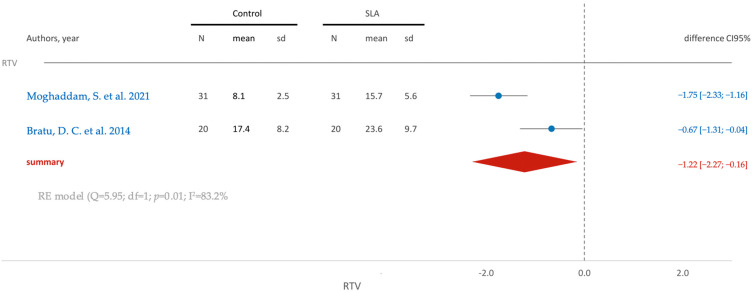
Comparison of RTV (removal torque value) in RCTs using SLA surface treatmen: blue color- results of individual studies; red color- meta-analysis result [12,18].

The calculated mean difference in removal torque between the control and SLA groups is −1.22, signifying a greater value in the SLA group. Notably, the observed difference attains statistical significance, as evident from the confidence interval (Figure 2).

**Figure 3:**
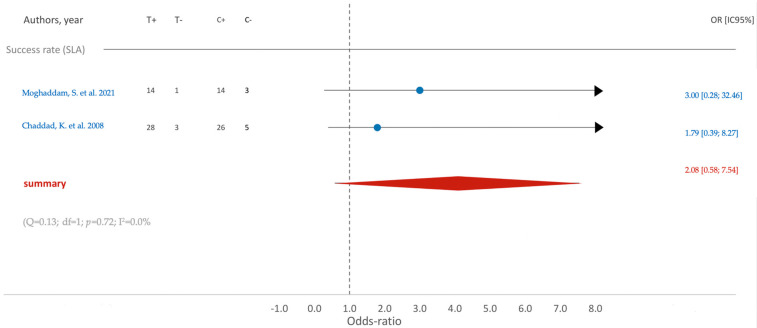
Comparison of SR (success rate) in RCTs using SLA surface treatment: blue color- results of individual studies; red color- meta-analysis result [13,18].

Concerning the success rate, an inclination towards improvement is noted in the test group (SLA), characterized by an average odds ratio of 2.08. Nonetheless, statistical significance was not reached (CI95% [0.58; 7.54]) (Figure 3).

**Figure 4:**
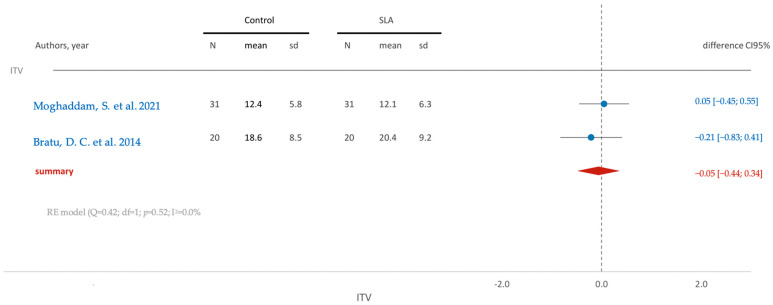
Comparison of ITV (insertion torque value) in RCTs using SLA surface treatment: blue color- results of individual studies; red color- meta-analysis result [12,18].

The insertion torque values exhibit similarity between the two groups, with a mean difference of −0.05 (95% CI [−0.44; 0.34]) (Figure 4).

**Figure 5:**
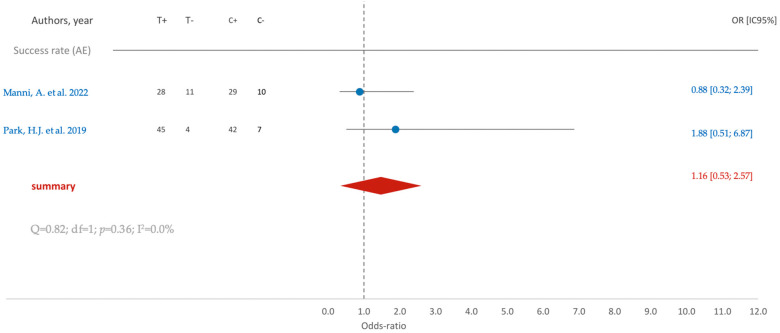
Comparison of SR (success rate) in RCTs using AE surface treatment: blue color- results of individual studies; red color- meta-analysis result [12,18].

Regarding the success rate associated with the acid-etching (AE) surface treatment, a subtle inclination toward improvement is observable, as indicated by an average odds ratio of 1.16. However, the confidence interval (95% CI [0.53; 2.57]) reveals a lack of statistical significance (Figure 5).

#### 3.5.2. In Vivo Studies

**Figure 6:**
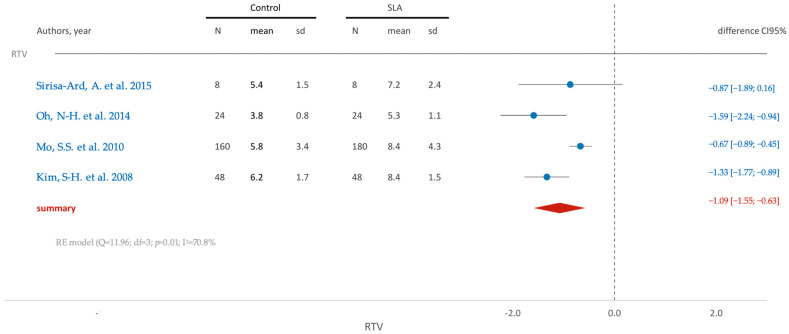
Comparison of RTV (removal torque value) using SLA surface treatment: blue color- results of individual studies; red color- meta-analysis result [71,74,84,88].

Figure 6 shows that similar to previous observations, the removal torque is higher in the test group (SLA), as is evident from the mean difference (−1.09), which achieves statistical significance, as affirmed by the confidence interval (95% CI [−1.55; −0.63]).

**Figure 7:**
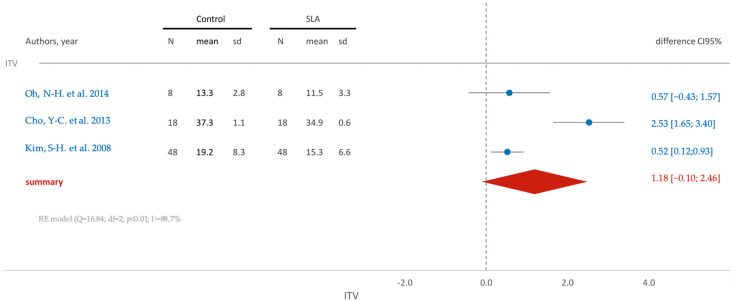
Comparison of ITV (insertion torque value) using SLA surface treatment: blue color- results of individual studies; red color- meta-analysis result [71,80,84].

The insertion torque within the SLA group, on average, appears slightly smaller (1.18) than that in the control group (Figure 7). However, upon considering the confidence interval (95% CI [−0.10; 2.46]), it is appropriate to conclude that there are no statistically significant differences between the groups.

**Figure 8:**
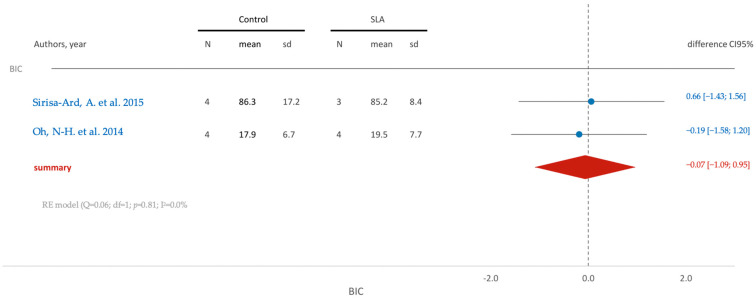
Comparison of BIC (bone contact interface percentage) using SLA surface treatment: blue color- results of individual studies; red color- meta-analysis result [84,88].

Figure 8 illustrates that there are no statistically significant differences (95% CI [−1.09; 0.95]) between the control and SLA groups concerning the bone contact interface.

**Figure 9:**
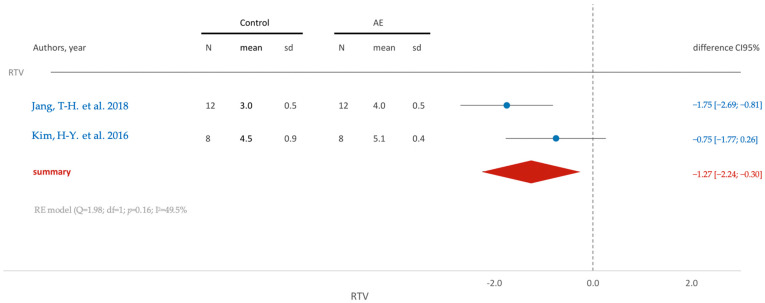
Comparison of RTV (removal torque value) using AE surface treatment: blue color- results of individual studies; red color- meta-analysis result [49,99].

For the acid-etching (AE) surface treatment, a parallel trend in removal torque is noted, mirroring that of the SLA group (Figure 9). The average removal torque value (RTV) is higher in the AE group, with statistically significant differences observed (95% CI [−2.24; −0.30]).

## 4. Discussion

The main differences between orthodontic miniscrews and conventional implants are the size, purpose, positioning, stability and duration of use. Orthodontic miniscrews are typically removed after they have served their purpose in orthodontic treatment, once the desired tooth movement has been achieved. Conventional implants are intended to create a direct structural and functional connection between living bone and the surface of a load-bearing artificial implant, providing long-term stable support for dental restorations [118]. As orthodontic miniscrews are designed for temporary use, they may not require as high a degree of stability as conventional implants [2,3]. Therefore, the complete osseointegration of TADs is a disadvantage that complicates the removal process; most of these devices are manufactured with a smooth surface, thus minimizing the development of bone growth and promoting soft tissue fixation under normal conditions [4]. Primary stability is obtained through mechanical retention of the screw in the bone, which depends on the design of the screw, bone properties and placement technique. On the other hand, secondary stability occurs due to the biological union of the screw with the surrounding bone and depends on bone characteristics, implant surface and bone turnover (cortical versus medullary bone). Over time, secondary stability increases while primary stability decreases [2,3]. Surface treatments on miniscrews can promote effective integration with the surrounding tissue, thus increasing the stability and longevity. Therefore, this systematic review aimed to summarize the surface treatments available on miniscrews in order to understand which ones could be adopted in the clinic and to compare their effectiveness in improving the outcomes proposed in the methods. A previous study, which included a meta-analysis of 14 studies, attempted to answer this question [12]. However, the results of this study should be assessed with caution as it presented several limitations in its methodology, namely: heterogeneity and poor quality of the animal studies included; observed faults in terms of methodology, such as the assessment of only one outcome; lack of coverage of in vitro and ex vivo studies; and the fact that PRISMA guidelines were not followed. Therefore, this review followed PRISMA guidelines in order to overcome the limitations previously described.

The SLA surface treatment was tested in all the types of studies reviewed. It involves the use of a beam of 100 to 500 µm aluminum oxide particles at constant pressure, followed by cleaning and then acid-etching [21,37,116]. Its aim is to increase surface roughness, promoting mechanical retention and greater integration into the underlying bone through greater fibroblast differentiation and proliferation [21]. This, in turn, allows for greater cell adhesion and protein absorption [21]. A previous study concluded that the action of SLA is related to improved cell adhesion to the surface in both healthy and diabetic individuals, the latter of which generally present with alterations in terms of bone metabolism [87]. This result was obtained through the excellent wettability of all the biological processes that derive from it, such as the increase in the exposed surface of the implant and the increase in bone–implant contact [22].

Acid-etching of surfaces, as shown in clinical studies included in our review, has been investigated through immersion in various acids in solution, such as nitric [102], sulfuric [20,93], phosphoric [39], hydrofluoric [28] and hydrochloric acid [38,50,93,102]. No studies were included that compared the effectiveness of the various types of acid. Two different studies have observed that surface acid-etching creates higher insertion torque values than untreated controls [93,117]. Fernandes et al. emphasized that insertion torque values are usually higher than removal torque values due to the predominance of compressive forces on insertion, which disperse with healing, reducing stability to a stable point [20]. With regard to the success rate of this treatment, Park et al. showed that although there is an increase in the number of successful miniscrews, this difference is not significant. These results are in line with those found in the literature on dental implants [117].

Additionally, some studies, mostly in vitro and in vivo, have proposed oxidative anodization protocols. This treatment is possible through immersing the miniscrew in an electrolytic solution at a constant voltage, promoting a potentiostatic system that creates a nanostructured titanium oxide matrix [90]. The anodization process can be carried out in one [105,109,119] or two steps [90,95,99], opening pores and depositing oxides, which provides the surface with nanoporosity that allows fibroblast proliferation [105]. These cells adhere to both flat and rough sites, confirming biocompatibility with the formation of dense bone tissue [105]. The data are in agreement with the systematic review and meta-analysis by Nagay et al., which aimed to understand the clinical efficacy of implants subjected to anodization used in different implant-supported prosthodontic solutions. It was concluded that the use of anodized implants as a form of prosthetic rehabilitation support is safe, but this method does not increase the effectiveness of the procedure [120].

APH treatment involves the use of anodization, followed by pre-calcification to incorporate calcium phosphate and hydroxyapatite, and heating. The results of this protocol are more favorable than those of anodization, with higher values for removal torque and bone formation. It is suggested that hydroxyapatite formation is accelerated, which is crucial for achieving stability in clinical cases of lower quality bone [44]. However, the risk of bias in interpreting the results must be taken into account. In fact, the authors failed to promote conditions for the correct randomization of the interventions, as well as the blinding of the participants and the evaluators of the outcomes.

Using energy-radiating devices, it is possible to perform photofunctionalization using ultraviolet light [14,49,92,98,106], photobiomodulation using low-intensity lasers [51,81,84,89,114,115] and diode light emission [18,82,104].

In photofunctionalization using UV light, the miniscrew is placed in an ultraviolet chamber, such as TheraBeam SuperOsseo, for 12 to 15 min. The radiation imparts superhydrophilicity to the surface, with increased stability and improved values for mobility, bone contact, resistance to lateral pressure and removal torque [14,49,92,98,106]. In addition, a systematic review and meta-analysis by Dini et al. showed similar results regarding the osseointegration and stability of conventional dental implants subjected to photofunctionalization using ultraviolet light in in vivo models. There was an improvement in osseointegration after the initial healing period through an increase in measured bone contact and cohesion strength, although the high risk of bias of the studies included in this publication, as well as the limited inclusion of study designs (animal models), should be noted [121].

Photobiomodulation can be carried out by laser emission at low wavelengths (635 to 1064 nm). Flieger et al. and Matys et al. used 635 nm and 808 nm lasers, respectively, and assessed stability and pain perception. They observed lower mobility values in the treated groups and no significant differences in the pain perception questionnaires [114,115]. However, the interpretation of pain perception in these studies raises some concerns when assessing the risk of bias, due to its subjectivity.

With regard to sterilization procedures, it was found that they do not lead to a decrease in stability due to previous use or sterilization [43], nor do they have a pronounced effect on fracture resistance [35]. The manufacturer of the miniscrew is the most differentiating factor in resistance [35]. Dry heat sterilization, which interfered with the mechanical properties of miniscrews, is noteworthy [63].

Regarding the meta-analysis, it was only possible to carry out this analysis for two surface treatments: SLA and acid-etching. The results of the meta-analysis found that both methods present enhanced attachment of the miniscrews, suggesting a better result for SLA than for acid-etching (odds ratio for the success rate 2.08 and 1.16, respectively). Despite the insertion torque in SLA treatment showing that the surface treatment of the miniscrews likely does not exert a noticeable impact on the initial attachment difficulty to the bone, the outcome of in vivo studies about the removal torque is higher in the SLA group, reinforcing the consistency of findings with prior results derived from RCT studies.

Reflecting on the large number of in vivo and in vitro studies that have been reviewed, although they reach conclusions that are mostly in agreement, they incur bias associated with blinding and allocation, as well as randomization. It is therefore recognized that the experience of the researchers, clinicians and collaborators involved in carrying out the different studies may have influenced the assessment of the results created. Future studies should evaluate the effectiveness of surface treatments with other variables such as screw designs, surface treatment protocols (e.g., acid concentration, composition, treatment time), implantation sites and animal models that can affect RTV, ITV and BIC.

## 5. Conclusions

Miniscrew surface treatments such as acid-etching; sandblasting, large-grit and acid-etching; photofunctionalization with ultraviolet light; and photobiomodulation were tested following well-defined and reliable protocols for increasing stability and the success of orthodontic treatment. These treatments can be applied in clinical orthodontic practice to enhance the effectiveness of miniscrews. Techniques such as oxidative anodization, anodization with pre-calcification and heat treatment, as well as deposition of chemical compounds, should be studied further, preferably in randomized, controlled clinical studies.

## Figures and Tables

**Table 1:** Summary of advantages and disadvantages of the most-cited surface treatments.

Surface Treatment	Advantages	Disadvantages
Sandblasting, large-grit and acid-etching [15,16]	Provides improved secondary stability. Success rate appears to increase with the use of SLA over machined miniscrews. Greater torque values achieved than with machined surfaces, due to increased surface area by roughness. Creation of insertion torque values over 15 Ncm may allow for better success.	Findings only pertain to immediate loading; more studies needed. The increase in success rate is not always statistically significant. Surgeon-reported ease of use was significantly different between machined and SLA-treated miniscrews, favoring the machined technique.
Acid-etching [17]	Higher success rate and primary stability. Facilitates osteogenic cell and blood cell retention. It allows for cell migration, improving the biocompatibility and stability of titanium miniscrews.	Findings not always statistically significant.
Photofunctionalization with UV-light [14]	UV light converts miniscrew surface from hydrophilic to superhydrophilic and improves contact between the bone and miniscrew. No changes in biocompatibility, no adverse reaction recorded to date.	This technique did not enhance the surface elements indicative of improved osteogenic potential.
Photobiomodulation with LED [18]	LED can accelerate orthodontic tooth movement and has a positive effect on stability, improving it over time.	No significant effect on IL-1β levels, indicating that it does not prevent peri-implantitis directly.

IL-1β: interleukin-1β; LED: light-emitting diode; UV: ultraviolet light.

**Table 2:** Summary of extrapolated data from included in vitro studies.

Author, Year	Sample	Type and Sample Size	Surface Treatment (Type, Time, Dose and Protocol)	Experimental Group	Control Group	Period Follow-Up	Test Used to Evaluate Outcomes	Primary Outcome	Results	Conclusions
Mattos, CT et al., 2010 [29]	Porcine femur cortical bone	Ti_6_Al_4_V (SIN, Brazil), 8 mm × 1.4 mm, n = 40	n = 20 Autoclave sterilization: STERMAX, one cycle, 30 min, 121 °C, n = 30 Ms recovered from 19 orthodontic patients and cleaned ultrasonically (Maxiclean 1400A) in an enzymatic detergent solution.	n = 20, autoclave sterilization. n = 30, ultrasonic cleaning	n = 20, no treatment		SEM at 20 kV: surface morphology analysis; Fracture Torque Tests (Digital Measurement).	Stability and risk of fracture	SEM: recovered with surface showing signs of use and marks; corrosion and defects were absent in the sterilized and recovered groups, as in the control. Fracture torque: significant difference between control group and recovered group; recovered with greater variation in values.	Recovered Ms have altered surfaces and a greater variance of FTVs. Reusing these is not recommended. The use of new Ms sterilized by autoclave is a recommended practice.
Mattos, CT et al., 2011 [35]	Porcine cortical bone in segments	Ti_6_Al_4_V: Neodent, SIN and Titanium Fix; n = 100	Five groups, one of each type of Ms; n = 10 × 5. Sterilization in STERMAX autoclave, 30-min cycle at 121 °C.	n = 50 Autoclave sterilization	n = 50, new ones	-	Tests fracture torque digital.	Stability	The effect of autoclave sterilization is not a significant factor affecting the variance of results (*p* = 0.4113), unlike the manufacturer of the Ms (*p* < 0.0001), with the differences in stability observed being due to this factor.	Autoclave sterilization of Ms did not cause pronounced effects on their fracture resistance, with the manufacturer being the most responsible factor for differences.
Muguruma, T. et al., 2011 [36]	-	Teeth, Ti-6Al-4V, 12 mm × 1.4 mm; n = 25	n = 20; Ultrasonic cleaning in ethanol and dimethyl ketone + immersion in solutions of 450 ppm F (G1 and G3) or 900 ppm F (G2 and G4) for 1 and 24 h, respectively.	n = 5 × 4; G1: 0.1% NaF 1 h; G2: 0.2% NaF 1 h; G3: 0.1% NaF 24 h; G4: 0.2% NaF 24 h	n = 5, no treatment	1 and 24 h	Torsion test: torques and fracture angles.	Stability	Torsion test: greater variations in fracture angle between groups than in fracture torques (similar between groups), but without significant differences compared to controls.	Use of these solutions as mouthwashes should not cause a decrease in the stability of the Ms and their clinical performance.
Cho, IS et al., 2012 [37]	Polyurethane sponge block	Ti_6_Al_4_V (Biomaterials Korea, Seoul, Republic of Korea), 8 mm × 1.45 mm, n = 54 = 18 + 36	(n = 18) SLA: 100 µm aluminum particles, 2 min, 2 bar + immersion 30% HCl, 60% H_2_SO_4_ and diluting solution. (n = 18) SLAO: SLA + oxidative anodizing (calcium acetate and β-glycerolphosphate) 3 min, 250 V.	n = 6 + 12 SLA; n = 6 + 12 SLAO	n = 6 + 12, no treatment	8 weeks	Digital measurement of ITV and RTV: analysis of maximum torque, total energy and peak energy.	Stability	Higher MITV in control (*p* < 0.01), but without differences in insertion TEV and NPE, or in all removal values.	SLAO treatment can be an effective way to reduce the damage due to the insertion of Ms, both in the tissue and in the device, and can also improve its stability.
Galli, C. et al., 2012 [38]	MC3T3 cells mouse	Titanium discs grade IV and grade V (HDC Company, Sarcedo, Italy) 10 mm × 1.5 mm, n = 6	Grade IV AE with hydrochloric acid. Grade V: AO by electrochemical protocol.	Grade IV G1: AE; G2: AE + calcium phosphate. Grade V: G1: AO; G2: AO + calcium phosphate	n = 2, without treatment in both grades	3 days	SEM:Cellular and surface morphology; Cell viability and organization analysis; PCR: expression analysis genetics.	Stability	Greater cell proliferation on smooth surfaces in grade IV compared to rough surfaces, and without differences in grade V. Calcium phosphate in grade IV increased the amount of messenger RNA for osteocalcin and alkaline phosphatase. Greater osteoblastic markers on grade V control surfaces than on rough ones, similar to grade IV titanium acid conditioning.	Ti grade IV with calcium phosphate obtained higher differentiation value in vitro. Machined Ti grade V had good cell proliferation and bone matrix synthesis, as well as greater expression of differentiating markers, which makes it an option for orthodontic Ms.
Noorollahian, S. et al., 2012 [39]	-	1.4 mm × 8 mm, 32: n = 16 new, n = 16 used for 3 years	H_2_O and drying + phosphoric acid gel 37% 1 mL + immersion in NaOCl 5.25 mL (10 mL) for 30 min.	NP1: irrigation + drying; P1: irrigation+ drying+ phosphoric acid + NaOCl (n = 16)	New Ms, C1: irrigation + drying (n = 16)	-	AAS: % calcium ion on the surface.	Amount Ca2 ion	NP1: 4.7 ppm; P1: 0.43 ppm; C: 0.02 ppm. NP1 significantly higher than other groups (*p* = 0.000), but P1 and C1 without significant differences between them.	Treatment reduces remaining tissue to manufacturing levels, allowing its use in processing used Ms.
Akyalcin, S. et al., 2013 [40]	-	Vector TAS, Aarhus, Dual-Top, Ortho Anchor 8 mm × 1.4 mm–1.5 mm (Aarhus system, American Orthodontics, Sheboygan, WI, USA); n = 120 = 30 × 4	n = 80; Autoclave sterilization (SciCan Statim 500) at 132 °C, 6 min. Insertion into synthetic bone blocks for all groups.	n = 20 per brand; G1 (n = 10): 5 sterilization cycles; G2 (n = 10): 10 sterilization cycles	n = 10 per brand; G0: 1 sterilization cycle	-	Maximum insertion torques and side displacement force.	Stability	ITVs: differences found between the four brands and between the three sterilization cycles and between them (*p* < 0.05). Lateral displacement: significantly affected by the brand, but not by the number of sterilization cycles; Arhus group with differences for the remaining brands (*p* < 0.05).	Depending on the manufacturer, each Ms has its own behavior. The values obtained for the stability of Ms indicate that sterilization up to and including 10 cycles does not affect the clinical stability.
Serra, G. et al., 2013 [41]	-	Prosthetic Systems Connection (Conexão, São Paulo, Brazil) 6 mm × 2 mm, n = 15	Surface nanostructuring (n Ti) by severe plastic deformation: grade 4 Ti pressed at 450 °C in longitudinal axis rotation (ECAP) + forging and drawing at 80% deformation + reheating at 300–350 °C for 1 h.	G2: Ti_6_Al_4_V; G3: nTi. Machined + clean + HNO3	G1: Pure Ti	-	Tensile strength test. Maximum Endurance Torque (MTR) Tests. SEM.	Stability	Elastic strength: G3 with fatigue resistance almost 80% greater than G1. MTR with increased values in G3 compared to control (*p* < 0.05), and no differences between G3 and G2. SEM: G3 with smooth morphology and transgranular fracture appearance; G3 with the roughest surface of all the groups.	nTi Ms bring together the biocompatibility of pure Titanium and the mechanical resistance of Ti_6_Al_4_V. They have greater torsional resistance than pure Ti and equal that of grade V titanium alloy.
Tozlu, M. et al., 2013 [42]	Bone model of bovine ilium (iliosacral joint), with cortical 0.5–2.5 mm, in strips	Ti-6Al-4V grade V cylindrical TM, Trimed, 9 mm × 1.6 μm; n = 48	n = 24 MI ring (MIR) device with four spikes which come into contact with the bone cortex when placing the ring on the Ms with a manual instrument, in a cavity in the Ms head, in order to support the ring.	n = 24 Mini Ti ring (Ti_6_Al_4_V grade V) MIR with spikes, fitted to the micro.	n = 24 without treatment	-	Mechanical test: anchoring strength; insertion and removal torque tests; measurement of cortical bone thickness.	Stability	AFR: experimental group showed significantly higher anchoring force values than the control (*p* < 0.001); the resistance of the control groups was influenced by the cortical thickness (*p* < 0.05). ITVs: significantly higher in the experimental groups (*p* < 0.01); higher insertion torque values the greater the thickness, for both groups (*p* < 0.01). RTVs: no significant influence of MIR.	The new MIR coupler device increased the anchoring force and insertion torque, therefore increasing the primary stability and resistance of the Ms under study. This device had no influence, however, on the removal torque and mobility values.
Estelita, S. et al., 2014 [43]	Porcine iliac bone in segmentsSR	n = 200	G1: insertion into high-density bone and removal; G2: initial protocol G1, ultrasonic cleaning 40 kHz, 25 °C, 20 min in detergent + irrigation with ultrasound + autoclave sterilization; G3: G1 protocol, but inclusion of sandblasting Al_2_O_3_, 90 µm, 60 psi, 20 min (n = 150).	G1, G2, G3	untreated high-density artificial bone inserted and removed (n = 50)	-	Tests fracture torque digital; sample weighing: mass loss analysis.	Stability	Similar fracture torques, not influenced by previous insertion into bone and sterilizing treatments. Ms diameter increases fracture torque with every 1 mm added, in any group. G3 with less weight compared to the others. Linear regression analysis showed that only the Ms diameter influences the variability of fracture torque, by 97%.	Ms do not suffer from decreased stability due to previous use or sterilization treatments. Sandblasting causes loss of mass, but without loss of torque. Differences of 0.1 mm in diameter influence removal torque, and therefore the stability of Ms.
Oh, EJ. et al., 2014 [44]	-	Ti_6_Al_4_V (Plates Kobe Steel Ltd., Japan) 10 × 10 × 2 mm	n = 16 APH treatment: TiO_2_ nanotubes in solution Glycerol/H_2_O/NH_4_F, 20 V, 1 h + cyclic calcification for incorporation of CaP and HA by repeated immersion in NaH_2_PO_4_(0.05 M), 80 °C and Ca(OH)_2_ (100 °C) 1 min/cycle for 30 cycles + heat treatment 500 °C, 2 h. Immersion in simulated body fluid (SBF).	APH: anodizing + pre-calcification + heat; AH: anodizing + heat	UT: no treatment	3 days, 3 and 6 weeks	FE-SEM; surface roughness test; microscopy assessment of hydrophilicity; measurement of removal torques; EDS/XRD; histological analysis of %BIC.	Stability and osteointegration	Morphology: APH group with an organized and compact arrangement of nanotubes, dense HA precipitate that fills all the empty spaces in the nanotubes. Bioactivity (EDS): APH group covered in HA protuberances; % Ca and P similar to HA, indicating good bioactivity.	APH treatment accelerated the formation of Hydroxyapatite, improved bone formation and presented surfaces with bioactivity and biocompatibility, which will allow an improvement in the initial stability of Ms. Indication for poor quality bone, where rapid healing and osseointegration is required.
Miyawaki, S. et al., 2015 [45]	-	Ti 6 mm × 1.6 mm, n = 4	SpikeAnchor, Ti_6_Al_4_V, two portions: portion that receives forces and portion with peaks that contact the cortical bone.	Implantation in the femur with SpikeAnchor	No Spike	4 weeks	Lateral displacement test: mechanical retention forces; visual analysis of device insertion.	Stability	The retention force was significantly greater in the experimental group at different displacements (*p* < 0.05). The smaller the displacement, the greater differences in the forces applied in the experiment. Spikes were implanted at 0.3 mm after application of compressive forces.	SpikeAnchor allowed automatic implantation over time into cortical bone. There was an increase in Ms stability of 3–5 times when compared to the experimental group and the control group.
Fleischmann, L. et al., 2015 [46]	Cells Osteoblast -likeMG-63	Discs Ti-6Al-4V 14.8 mm × 0.6 mm	G1: Discs coated in TiN with TiN plasma spray, 6 µm; G2: Discs coated with PTFE powder + oven heating = 40 µm PTFE.	G1: TiN plasma; G2: PTFE	G3: no treatment; G4: plastic cells	48, 120, 168 h	MTT test. Time-lapse microscopy Apoptosis test by flow cytometry; alkaline phosphatase activity and PCR for osteogenesis markers.	Stability	Viability/Proliferation: 168 h: PTFE and G4 groups showed greater cell viability than the other groups (*p* < 0.05). Cell behavior: at 12.5 h adhesion to G3 surfaces, 50% of cells to adhere in the PTFE group. Apoptosis: G1 and G2 have a lower apoptotic rate (*p* < 0.05) than G4. Gene expression: alkaline phosphatase and osteocalcin mRNA higher in the PTFE group than in G3 (*p* < 0.05). Osteoprotegerin: higher in PTFE than in G3 (*p* < 0.05).	Surface treatment with PTFE on titanium showed better biocompatibility, both in relation to the non-use of treatment and the use of TiN. It is suggested that this promotes osteointegration and hydrophilicity, with less microbial adhesion. More in vivo studies are needed.
Ganzorig, K. et al., 2015 [47]	MC3T3-E1 cells	33 mm discs (Nishimura Metal, Japan) Ti6Al4V and 1.6 mm × 1.5 mm SS, n = 140	LIPUS Osteotron_D IB, Ito Co; 1.5 MHz, 30 mW/cm^2^, pulse ratio 1:4; 24 h after insertion, 20 min/day in the right tibia (1×/15 min in the cells).	n = 80, LIPUS	n = 80, no treatment	0–14 days	Alkaline phosphatase test. Light microscopy; analysis of the presence of minerals; PCR.	Stability and osteogenesis	From day 3 to day 14, BIC gradually increased in all groups. LIPUS increased BD density, CBT and bone formation rate after insertion (*p* < 0.05). Increase in GLIPUS of ALP regulation in vitro on the third day (*p* < 0.05).	It is suggested that the application of LIPUS improves bone formation around titanium alloy and stainless steel Ms and may therefore improve their initial stability and success rate throughout orthodontic treatment.
Liang, Y. et al., 2015 [48]	-	Ti grade 4: 10 × 10 × 2 mm boards	Strontium (Sr) application: polishing + HCl + CaCl_2_ + ultrasonic cleaning in acetone, 10 min + H_2_O + drying + ECD (electrochemical deposition with platinum mesh).	n = 12 G1: without ECD, ovariectomy; n = 12 G2: ECD with Sr, ovariectomy	n = 12, no treatment + no ovariectomy	2 and 4 weeks	XRD: analysis of the chemical composition; FE-SEM.	Stability	XRD: coating with SrHPO_4_, with peak intensity higher than other crystals. FE-SEM: thickness 25 µm; morphology of lamellar crystals in clusters.	Strontium treatment is easily performed by ECD. This has a promoting effect on the osteointegration of Ms in animals with osteopenia and may be a new protocol in patients with osteoporosis.
Tabuchi, M. et al., 2015 [49]	Bone marrow cells from 8-week-old Sprague-Dawley rats	6 mm × 1.4 mm (Jeil Medical, Guro-gu, Republic of Korea), n = 12; Ti_6_Al_4_V discs (n = 18) 20 mm × 1.5 mm	Before implantation and placement in culture, the Ms and discs, respectively, were treated with ultraviolet light for 12 min with a TheraBeam Super Osseo device.	UV Light	No treatment	3 weeks	SEM; EDX; ELISA; CLSM: growth and cell adhesion; alkaline phosphatase activity; mechanical Tests.	Stability	Number of osteoblasts adhered to the experimental surface at 3 and 24 h is significantly higher than in the control (*p* < 0.05); experimental group with higher expression of vinculin and actin (*p* < 0.001 and *p* < 0.01, respectively); positive area for alkaline phosphatase (ALP) 80% greater in the functionalized groups, and equally in its activity and calcium deposition (*p* < 0.001).	The displacement of photofunctionalized Ms was 30–40% less than untreated ones. It is suggested that photofunctionalization increases the bioactivity of the Ms under study, as well as their anchoring capacity and stability, without changing the mechanical morphology itself.
Yadav, S. et al., 2015 [50]	-	Circular discs 3 mm × 3 mm Ti6Al4V grade V (Dentaurum Co., Ispringen, Germany)	G2 (n = 32): AE 0.11 mol/L HCl 65 °C for 20 min + oven drying for 24 h; G3 (n = 32): gritblasting with 25–50 µm alumina; G4 (n = 32): gritblasting with alumina 25–50 µm + AE 0.11 mol/L HCl 65 °C for 20 min + oven drying for 24 h.	G2: AE (n = 32); G3: gritblasting (n = 32); G4: gritblasting + acid-etching (n = 32)	n = 32, G1: no treatment	8 weeks	Optical profilometer: surface roughness; goniometer: contact angle measurement; removal torque test; histological analysis (toluidine blue): BIC.	Biological integration	Surfaces: G1: irregularities in 1 direction; G2: thin and hard surface with elevations and depressions; G3: highly irregular surface and cavities; G4: uniform surface, with smaller cavities than G3. Hardness: G3 < G4. Wettability: G4 lower with blood and sodium chloride (*p* < 0.0002); G2 and G3 smaller than G1 (*p* < 0.0003); for DMSO and water, there were differences between all groups. RTV: no significant interaction between bone type and Ms surface; G4 with significantly greater torque than G3, G2 and G1. Contact: significantly higher in G4 than G3, G2 and G1.	Surface hardness was shown to be higher in gritblasted Ms than in others, following this coupled with acid-etching with HCl. Contact with liquids was greater for the control group and lower for gritblasted with conditioning. The removal torque in both the tibia and femur was greater in the gritblasted with conditioning group. Greater bone/Ms contact on rougher surfaces than on machined surfaces alone.
Fleischmann, L. et al., 2015 [46]	Cells Osteoblast -likeMG-63	Discs Ti_6_Al_4_V 14.8 mm × 0.6 mm	G1: Discs coated in TiN with TiN plasma spray, 6 µm; G2: Discs coated with PTFE powder + oven heating = 40 µm PTFE.	G1: TiN plasma; G2: PTFE	G3: no treatment; G4: plastic cells	48, 120, 168 h	MTT test. Time-lapse microscopy Apoptosis test by flow cytometry; alkaline phosphatase activity and PCR for osteogenesis markers.	Stability	Viability/Proliferation: 168 h: PTFE and G4 groups showed greater cell viability than the other groups (*p* < 0.05). Cell behavior: at 12.5 h adhesion to G3 surfaces, 50% of cells to adhere in the PTFE group. Apoptosis: G1 and G2 have a lower apoptotic rate (*p* < 0.05) than G4. Gene expression: alkaline phosphatase and osteocalcin mRNA higher in the PTFE group than in G3 (*p* < 0.05). Osteoprotegerin: higher in PTFE than in G3 (*p* < 0.05).	Surface treatment with PTFE on titanium showed better biocompatibility, both in relation to the non-use of treatment and the use of TiN. It is suggested that this promotes osteointegration and hydrophilicity, with less microbial adhesion. More in vivo studies are needed.
Espinar-Escalona, E. et al., 2016 [28]	-	Ti pure (HDC Company, Sarcedo, Italy), 9 mm × 2 mmn = 20	AE: immersion in 0.35 M HF, 15 s, 25 °C. Gritblasting: 600 µm alumina particles, 0.25 MPa until roughness saturation. GBA: combination of previous techniques. Acetone cleaning protocol, 15 min + H_2_O + drying in N_2._	n = 5 AE: conditioning acid; n = 5 GB—alumina beam; n = 5 GBA—AE + GB	n = 5, no treatment	10 weeks	FE-SEM: analysis of the O/M interface. Manual measurement of removal torque. Analysis of %BIC.	Stability	Roughness of GB and GBA significantly higher than control and AE. GB and GBA significantly more hydrophobic than the rest. %BIC: GBA 79% and GB 75% (*p* < 0.05), AE 26% and control with 19%. No differences in removal torque between AE and control groups, but higher values for GB and GBA. Greater roughness and wettability in treatments than in control, leading to higher %BIC and higher RTV.	Surface treatments on Ms such as AE, GB and GBA are effective in modifying the surface and improving osseointegration and stability of the device. These create removal torques that do not compromise stability and do not promote fractures. Wettability was the parameter that showed the most influence on torque.
Kang, H.K. et al., 2016 [51]	-	316 SS stainless steel 6 mm × 1.2–1.3 mm, n = 48	Nd-YAG 1064 nm treatment Q-switched.	n = 12, Nd-YAG laser Q-switched	n = 12, no treatment	0 and 8 weeks	(ZeGage): surface roughness measurement x3; SEM; fracture torque tests (digital measurement).	Stability	All Ms with mobility <1 mm. No flaws. Surface roughness: higher values in the treated group (*p* < 0.05), triple increase; No differences in fracture torque and 2D and 3D BIC between groups.	Surface roughness increased by more than three times due to surface reactions created by the laser. There was no increase in fracture resistance, despite an increase in surface roughness.
Kim, H.Y. et al., 2016 [52]	-	OSSH1606 (Osstem Implant, Seul, Republic of Korea) 1.6 mm × 6 mm Ti6Al4V, n = 150	G1: hydrochloric and nitric AE; G2: RBM, calcium phosphate beam and acid wash—removed from in vivo experiment due to fractures at placement; G3: hybrid: 75 µm calcium phosphate beam, except for cutting one-third and acid washing.	G1, G2 and G3	G0: no treatment	1, 2, 4 and 8 weeks	Insertion and removal torque tests; optical microscopy; SEM: surface changes; EDS: quantitative analysis of surface composition.	Stability	Cutting capacity: more superficial insertion in the RBM group than other groups (*p* < 0.05). Osseointegration: at 4 weeks, the removal torque in the control group decreased significantly, but was increased in G1 (*p* < 0.05 in both); G4 with increased values at 2 weeks, and with higher values compared to the other Gs at 8 weeks (*p* < 0.05). There was infiltration of calcium and phosphorus on the surface; bone was detected in the G4 group.	Partial/hybrid RBM group with greater stability compared to the other groups, without a reduction in cutting capacity.
Fernandes, D.J. et al., 2017 [20]	-	Ti_6_Al_4_V Discs (ASTM grade V), 6.35 mm	Polishing with 0.05 µm alumina + washing with acetone, alcohol and water. AE: (HNO_3_ + H_2_O + H_2_SO_4_) under magnetic stirring + HNO_3._	n = 24 AE (1, 4 and 8 s)	n = 24, no treatment (1, 4 and 8 s)	1, 4 and 8 weeks	FE-SEM; SPM: thickness and roughness of the TiO_2_ layer. Goniometer; Panalytical X’Pert PRO: investigation of the crystal structure. XRF: surface composition.	Stability	No fractures or infections. Surface: intercommunicating micropores in the experimental group; micro and sub-microscopic roughness; TiO layer two greater in Ms with treatment, and with lower % of Al and V. Insertion and removal torque: higher values for the treated group; ITVs > RTVs. Histological analysis: dense Ca/P particles with proliferating osteoblasts at the O/M interface of the group treated at 4 weeks, new bone formed at 8 weeks. Blood analysis: % Al and V decreased in all follow-ups in the treated group.	Acid-etching treatment of the surface of Ms improves surface morphology and mechanical stability, with early signs of osseointegration. It also allowed a reduction in the release of Al and V ions.
Pop, S. et al., 2017 [53]	Synthetic pig bone, high density	Linkfrom MIS™ (MIS Implants Technologies, HaZafon, Israel) and Yesanchor (Orlus™, Ortholution, Seoul, Republic of Korea) both 1.6 × 8 mm, n = 100	G1 (n = 10): ultrasonic cleaning 40 kHz, 25 °C, double + autoclave 121°, 15 psi for 20 min; G2 (n = 10): chemical cleaning (37% phosphoric acid gel) 10 min + cleaning, drying and 5.25% NaOCl 30 min + cleaning + autoclave as G1; G3 (n = 10): insertion and removal protocol G1 + ultrasonic detergent cleaning 8 min + cleaning distilled water + sandblasting Al_2_O_3_-90 µm 60 psi + cleaning and ultrasonic bath 20 min + autoclave; G4 (n = 10): distilled water + autoclave.	n = 40, G1: ultrasonic cleaning and autoclave; G2: chemical cleaning + autoclave; G3: ultrasonic cleaning + sandblasting + autoclave; G4: cleaning distilled water + autoclave	n = 10 new ones, unused, untreated	-	Maximum Insertion Torque Test.	Stability	Average ITV ranged: Link 22.40 Ncm—26.94 Ncm; Yesanchor 36.46 Ncm—42.37 Ncm. Significant differences between G0 and G4 (*p* = 0.0177), G2 and G3 (*p* = 0.0402) and G3 and G4 (*p* = 0.0135), G3 with greater torque than G2 and G4; no differences between groups Between Link and Yesanchor groups, there were statistically significant differences (*p* < 0.001).	Differences in maximum insertion torque specific to the brands of Ms used were found. Different types of chemical and mechanical cleaning of Ms create variable effects on torque values, differences that are more pronounced in Link Ms. More studies are needed in order to find other modifications and their influence on other parameters, such as surface topography, or fracture and removal torque.
Tejani, H. et al., 2017 [54]	-	Teeth, Ti-6Al-4V, n = 120	n = 2 × 30. New group and 11-year-old group; sterilization with autoclave + UV light treatment with 5 × 8 W bactericidal lamp in a 254 nm tube, 12 min, with carbon deposition measurements following both treatments.	n = 90; Sterilization + UV Light G1: 6 years of archive; G2: 9 years of archive; G3: 11 years of archive	n = 30; G0. Autoclave + UV Light, new manufacturing	-	XPS: carbon content of tested titanium surfaces; water contact angle test.	Stability	Archive time: the longer it is, the higher the carbon content. Sterilization: increase in carbon content in G0. UV light: significantly decontaminated surfaces (lower % carbon); G4: with differences in carbon load due to UV light and not autoclave sterilization; increased hydrophilicity of samples by UV treatment, but without increased cellular activity.	Ms with a longer archive time will have a greater amount of carbon contamination on their surface. Steam sterilization with an autoclave increased the carbon content on the surface. UV light reduced the carbon content. However, it did not modify the osteoblastic differentiation.
Kaci, N. et al., 2018 [55]	-	Ti grade 23, 8 mm × 2 mm, n = 52; CPT Pure Ti 10 mm × 2 mm	Reuse of Ms for four different periods: G1 0 days of use; G2 2 months of use; G3 1 year of use; G4 14 months of use. Reinsertion in the upper posterior region.	G1: n = 6; G2: n = 6; G3 n = 20; G4 n = 20	G0: CPT new 10 mm × 2 mm	-	Image polarized optics: surface analysis. Torsional Mechanical Tests: Fracture Resistance.	Stability and fracture resistance	Surface characteristics for minor uses revealed no defects at the micron level, and with fracture torques of around 53 N/cm^2^. G3 and G4 with evident surface changes, mainly where there was an interface with the gingiva, and with fracture torque of 42–39 N/cm^2^, respectively.	Grade 23 titanium is a good compromise between pure titanium and stainless steel. Mechanically, the reuse of Ms that have been used for 0–2 months is possible, but it is not recommended for those with longer use, as there is a decrease in their resistance.
Pop, S. et al., 2018 [56]	Pork jaw; artificial bone 1 cm^3^	YesAnchor (Orlus, Ortholution, Seoul, Republic of Korea) 8 mm × 1.6 mm, n = 50	Ultrasonic cleaning + autoclave sterilization; cleaning with 37% phosphoric gel, 10 min + immersion in NaOCl 5.25%, 30 min + autoclave sterilization; insertion and removal + cleaning + Al_2_O_3_ +autoclave sterilization; insertion and removal + cleaning H_2_O + sterilization in autoclave. All groups subsequently inserted into artificial bone (including YA0).	n = 10 × 4 YA1; YA2; YA3; YA4	n = 10, YA0: new, without treatment or insertion	-	Insertion energy measurement. Microscopy: analysis of the degree of morphology change.	Stability	YA1 group with the highest average value of insertion energy. Only significant differences in maximum insertion energy between YA1 and YA3 (*p* = 0.04).	No significant differences in the behavior of Ms in groups YA1, YA3 and YA4. Decrease in total insertion energy in the YA3 group due to the use of sandblasting. Sterilization by autoclave followed by cleaning with distilled water did not promote total elimination of the organic tissue remaining on the surface of the YA4 group. Presence of chemical corrosion if used chemical cleaning.
Miyawaki, S. et al., 2015 [45]	-	Ti 6 mm × 1.6 mm, n = 4	SpikeAnchor, Ti_6_Al_4_V, two portions: portion that receives forces and portion with peaks that contact the cortical bone.	Implantation in the femur with SpikeAnchor	No Spike	4 weeks	Lateral displacement test: mechanical retention forces; visual analysis of device insertion.	Stability	The retention force was significantly greater in the experimental group at different displacements (*p* < 0.05). The smaller the displacement, the greater differences in the forces applied in the experiment. Spikes were implanted at 0.3 mm after application of compressive forces.	SpikeAnchor allowed automatic implantation over time into cortical bone. There was an increase in Ms stability of 3–5 times when compared to the experimental group and the control group.
Hergel, C. et al., 2019 [57]	Sawbone Artificial Bone	Dual-Top 8 mm × 1.6 mm (Jeil Medical, Republic of Korea); Ortho-Easy 8 mm × 1.7 mm (FORESTADENT, Germany), n = 140 = 70 × 2	n = 60 + 60. Insertion into Sawbone artificial bone, followed by removal. Ultrasonic cleaning 30 min, 1LH_2_O, 5 mL Endozyme + sterilization in an autoclave at 135 °C. 10 min + Statim 7000 drying, 55 min.	G2: insertion + Sterilization + insertion; G3: (insert + autoclave) × 2 + insertion	n = 10 + 10; without insertion and treatment	1 month	ITVs and RTVs. Vertical and horizontal resistance Tests. SEM; Fracture Torque Tests.	Stability	Significantly higher MITV in G1 (*p* < 0.05). No differences were found in MRTVS and the remaining VH Tests and torsional strength. SEM: atrophy of the coils of the Ms used was detected, + in the apical region; oxidized layer disappeared in some places in G2 and 3.	Although some wear and atrophy of the Ms used has been proven, their primary stability and fracture torque values did not show significant differences after a second insertion.
Iodice, G. et al., 2019 [58]	Osteoblast cells -like Osteosarc oma Saos-2	Grade V Titanium Plates Orthoeasy^®^ (FORESTADENT, Germany) 10 mm × 2 m; n = 272 (68/type)	AO treatment (different thicknesses of titanium oxide on the surface of titanium plates—pink, gold and rosé groups).	Thickness TiO_2_ n = 68 each: G.pink 40–50 nm; G.golden 130 nm; G. rose 140 nm	n = 68, Gray group: without layer TiO_2_	12, 24, 40 and 48 h	CLSM: cell growth, Live/Dead™ Viability/Cytot oxicity Kit: cell viability analysis; Hoechst and α-tubulin staining.	Stability	Growth: higher in Procollagen I control (*p* = 0.019), Rosé with higher concentrations. Viability: differences in live cells (*p* = 0.016), but absence in dead cells. Migration: differences in cell-free areas at 12 h, 24 h and 40 h, with no free areas at 48 h, lower values in control and with larger ones in rosé at 24 h and 40 h, therefore showing faster and slower migration, respectively.	Anodization to obtain TiO_2_ produces minor effects on cell viability, although greater in the initial population times. There is no clear relationship between thickness and cellular response to Ms.
Jongwannasiri, C. et al., 2019 [59]	Porcine mandibular bone	Ti6Al4V (Osstem Implant, Seul, Republic of Korea), 10 mm × 1.8 mm	DLC (diamond-like carbon): thin film of diamond carbon reinforced by Fluorine or Silica, through a mixture of gases containing C_2_H_2_, CF_4_ and Si(CH_3_)_4_ or tetramethylsilane(TMS) in a vacuum chamber, 2 Pa, 5 kV.	G DLC, without F or Si, G F-DLC, G Si-DLC	No treatment	-	Friction tests: dry air and ambient air at 20 °C. Modified Dulbecco method. Measurement of torque insertion.	Stability and healing	0%RH and 40%RH, F-DLC influenced the friction coefficients and Si-DLC significantly influenced the morphology of the carbon films (less friction the higher Si), these being less cytotoxic than in the other groups. Lower insertion torques for groups F and Si.	These treatments can be considered to improve the performance of orthodontic Ms.
Kim, H.Y. et al., 2016 [52]	-	OSSH1606 (Osstem Implant, Seul, Republic of Korea), 1.6 mm × 6 mm Ti6Al4V, n = 150	G1: hydrochloric and nitric AE; G2: RBM, calcium phosphate beam and acid wash—removed from in vivo experiment due to fractures at placement; G3: hybrid: 75 µm calcium phosphate beam, except for cutting one-third and acid washing.	G1, G2 and G3	G0: no treatment	1, 2, 4 and 8 weeks	Insertion and removal torque tests; optical microscopy; SEM: surface changes; EDS: quantitative analysis of surface composition.	Stability	Cutting capacity: more superficial insertion in the RBM group than other groups (*p* < 0.05). Osseointegration: at 4 weeks, the removal torque in the control group decreased significantly, but was increased in G1 (*p* < 0.05 in both); G4 with increased values at 2 weeks, and with higher values compared to the other Gs at 8 weeks (*p* < 0.05). There was infiltration of calcium and phosphorus on the surface; bone was detected in the G4 group.	Partial/hybrid RBM group with greater stability compared to the other groups, without a reduction in cutting capacity.
Ly, N. et al., 2019 [60]	MC3T3-E1 cells of mouse	Ti_6_Al_4_V AbsoAnchor (DENTOS Inc., Daegu, Republic of Korea) + TI disks (Ti-6Al-4V)	Chitosan covalent bond; aminofunctionalization (APTES—PM, 221.37 g/mol) in deionized water, 4 h + cleaning with 70% ethanol and drying + grafting with spacer: succinic acid (SA—PM, 118.09 g/mol) or polyacrylic acid (AA—PM, 150,000 g/mol), 6 h + drying + binding of 0.5% chitosan (low molecular weight, 50,000 g/mol, ADI, 75%) with 2% acetic acid, 8 h + saline solution wash.	Treatment Covalent bonding of chitosan (succinic acid spacer or polyacrylic acid)	No treatment	24 and 72 h	FE-SEM: cell adhesion test; ELISA: cell proliferation test; CLSM: feasibility test; absorbance test at 595 nm: biofilm formation.	Stability and cellular response	Lower contact angle when using succinic acid. Greater contact with samples with chitosan and succinic acid. FE-SEM: at 72 h greater cell adhesion in the SA experimental group than in the control; at 24 h, no significant difference; at 72 h, significant greater proliferation with the use of chitosan (*p* < 0.05). CLSM: at 24 h similar viability between control and SA-CH; at 72 h, experimental group has no dead cells and an increase in live cells. Antibacterial activity: effective reduction of the biofilm created in both microbial samples through the action of chitosan (and SA) (*p* < 0.05).	The use of succinic acid spacer was more advantageous than polyacrylic acid. Surfaces modified by chitosan, with SA spacer, presented hydrophilic nanostructures, which promoted cell adhesion, proliferation and cell viability, as well as reduction of biofilms by *S. mutans* and *S. sobrinus* by 53% and 31%, respectively. It is suggested that treatment with chitosan may promote the stability and antibacterial properties.
Oga, Y. et al., 2019 [61]	-	Dual-Top (Jeil Medical, Republic of Korea), 6 mm × 1.6 mm + auxiliary built-in device Ti_6_Al_4_V, ASTM F136-96, PCT + Durometer silicone rings; n = 42	n = 22 Ti auxiliary built-in device Ti_6_Al_4_V with two portions: capture of compressive forces + three peaks inserted into the bone cortex.	n = 11 4 s; n = 11 8 s; placement with assistive device	n = 9 4 s; n = 11 8 s; No treatment	4 and 8 weeks	Lateral displacement test with compression test machine: mechanical retention analysis.	Stability	Cortical thickness: at 4 s, auxiliary group (GA) 1.33 m and control 1.41 mm; at 8 s, GA 1.41 mm and control 1.44 mm (both follow-ups *p* > 0.05). Peak insertion depth: GA 0.28 mm 4 s and 0.37 mm 8 s. Lateral displacement: GA had significant effectsts on lateral displacement (*p* < 0.01), not taking time into account. Retention force: GA in 4 s and 8 s greater than controls in all displacements.	The automatic anchoring auxiliary device coupled to the Ms increased its stability, on average, by 1.6 to 2.8×. It may be possible to allow the use of Ms smaller in length and diameter, fundamental characteristics in substrates that are more difficult to insert.
Pavlic, A. et al., 2019 [62]	Lactobacillus us reuteri	316 Stainless steel (SS) Ti grade V OrthoEasy (FORESTADENT, Germany); Ti grade 23 (Unitec 3M, Monrovia, CA, USA), n = 12 × 5 = 60	Immersion in: artificial saliva (AS) 1.5 g/L KCl, 1.5 g/L NaHCO_3_, 0.5 g/L NaH_2_PO_4_ × H_2_O, 0.5 g/L KSCN, 0.9 g/L lactic acid, pH 4.8; probiotic bacteria Lactobacillus reuteridiluted in AS ph4.8 1:1 in 30 mL; Oral antiseptic chlorhexidine-digloconate 0.05% with 0.05% sodium fluoride with AS pH 4.8 of 1:1.	G1: artificial saliva; G2: Probiotic; G3: Chlorhexidine. For each type of microimplant five times	No treatment	28 days	AFM: roughness of surface; Vickers method: microhardness analysis. SEM: qualitative analysis of topography.	Stability	Roughness: lower in untreated SS than in both Ti Ms (*p* < 0.001); lower in Ti grade 5 than grade 23 for average roughness parameter (*p* < 0.002); Ti grade 23 with greater roughness, except in chlorhexidine; Ti grade 5 with greater roughness in probiotics than in other media. SS group loses chlorhexidine treatment; greater roughness in Ti grade 23 in saliva; marked corrosion in the Ti grade 5 probiotic group; less corrosion in group Ti grade 23 than in others. Hardness: equal between Ti groups, and lower in SS groups (*p* < 0.001), AS and probiotics provide similar hardness in all groups.	Grade 5 titanium Ms showed an increase in surface roughness in the presence of probiotics. SS show increase when treated with chlorhexidine. SA increased roughness only in grade 23 Ti. Guidance on the use of chlorhexidine in patients with titanium Ms and probiotics in SS.
Alavi, S. et al., 2020 [63]	-	Jeil (Jeil Medical, Republic of Korea); Hubit; 8 mm × 1.6 m, n = 72 = 6 × 12	G1: steam sterilization (MELAG autoclave, Euroklab) at 121 °C, 15 psi, 15 min; G2: sterilization by dry heat at 161 °C, 2 h.	n = 4 × 12 G1: steam sterilization with autoclave; G2: dry heat sterilization	n = 12 × 2; G = 0 without treatment	-	Insertion and fracture torque tests.	Stability	Jeil: differences in insertion torques between G2 and G0 (*p* < 0.001); differences between groups in fracture torque (*p* < 0.001). Hubit: no differences. There were significant differences between the torque values between the two manufacturers.	Steam sterilization did not have any adverse effect on the stability of Ms. Dry heat sterilization interfered with its mechanical properties.
Giri, M. et al., 2020 [64]	Bull femur	Ti (DENTOS Inc., Daegu, Republic of Korea); 8 mm/10 mm × 1.3 mm conical or cylindrical; n = 24	n = 20 Radio frequency current, Vacuum sputtering unit model BC-300, Yttrium stabilized Zirconia deposition.	n = 20 = 4 × 5 Zirconia in four groups	n = 4; You without treatment		ITVs tests; SEM: surface analysis; XRD: structure of the layer of zirconia.	Stability	Maximum ITV: no significant differences between experimental and control values; values in all groups quite similar.	Insertion changes increased cylindrical Ms, as well as those with greater length. Ms treated with coatingzirconia maintained its structural integrity.
Iwanami-Kadowaki, K. et al., 2021 [65]	Cells Osteoblast -likeMG-63	Grade II titanium plates (Kobe Steel, Ltd., Kobe, Japan), 20 × 50 × 0.5 mm^3^, n = 24	1 g modified Hap/Col powder + 100 mL 2-propanol suspension, 2 mL glycerol, 1–50 mg hydrated magnesium nitrate, 2 mL H_2_O + dispersion ultrasonic 10 min + voltage 20–60 V/cm 2–6 min applied to Ti plates (cathode) covered with stainless steel plates (anode) + cleaning with 2-propanol + drying.	Plates with hydroxyapatite and collagen treatment at different follow-ups	Untreated Ti board	1, 3, 5 and 7 days	Zeta potential and sedimentation analysis; SEM and laser microscopy; EDS; Tape test: adhesive strength of the coating.	Stability	Successful creation of surface treatment, and the thickness increases with treatment time and voltage and presence of cracks and fractures. Two min at 20 V seemed to be the protocol with the smoothest and flattest thickness. The existence of cracks did not influence the adhesion of the HAp/Col layer to the titanium. Cell viability was identical between the control and experimental groups (*p* < 0.01).	It was demonstrated that hydroxyapatite and collagen surface treatment could be achieved with controlled thickness and high adhesion strength on titanium surfaces. It also showed excellent biological properties.
Zogheib, T. et al., 2021 [66]	Rib of cow	Dual Top^®^ (Jeil Medical, Republic of Korea), Spider Screw^®^ (HDC Company, Sarcedo, Italy), Absoanchor ^®^ (DENTOS Inc., Daegu, Republic of Korea),Microdent^®^ (Barcelona, Espanha) 6–8 mm × 1.4–1.6 mm, n = 96	G1: (n = 12) OA with 0.1 mM/L H_2_SO_4_ at 5V for 20 min G2: (n = 12) Sandblasting (SB) by alumina projection 50-µm, 20 psi, 1 min, 1 cm distance, 45° angle + ultrasonic cleaning 5 min in acetone +drying; G3: (n = 12) Sandblasting G1 and AO (SBAO) with 0.1 mM/L H_2_SO_4_ at 5V for 20 min.	G1: AO (n = 8); G2: (n = 12) Sandblasting (SB); G3: (n = 12) Sandblasting and oxidative anodizing	SEM of n = 8 for surface analysis and G0: n = 4 untreated (one each brand)	-	SEM and EDX: surface and chemical composition; optical profilometer: surface roughness; Sessile drop method: contact angle static.	Stability	Control with irregular surface contaminants originating from materials inherent to production. Handling also showed other contaminantsSR. Higher surface roughness in the SB group, and higher contact angle in the SBAO group. Evidence of bone contamination with particles from the surface of microimplants is observed, which increases with preparation for clinical procedures.	Surface treatments increased surface roughness and the bone/Ms contact angle, which could promote osseointegration. The insertion and removal of these devices leaves contaminating particles in the bone. The use of gauze with 0.12% chlorhexidine is recommended when handling Ms. Required more studies.
Im, C. et al., 2022 [30]	MC3T3 pre-osteoblastic cells mouse	Ti–6Al–4V ELI Boards (Kobe Steel Ltd., Kobe, Japan) 20 mm × 10 mm × 1 mm; n = 18	AO (HNO_3_ + HF + H_2_O) 10 s + H_2_O+ 1.4 wt% NH4F) 20 V, 60 min + P: 0.5 vol% silicate, 5 min + drying 1 h + 20 × (0.05 M NaH_2_PO_4_ and Ca(OH)_2_, 90 °C, 1 min interval). Heat: electric furnace at 500 °C, increase 10 °C/min, 2 h.	AH: anodizing and heat treatment; APH: anodizing, pre-calcification and heat	UT: no treatment	2, 3 and 4 days	SBF tests: analysis of bioactivity by EDS; WST-8 test: cytotoxicity assessment.	Stability	Surface: AH—dense and aligned formation of nanotubes, with protrusions; APH—presence of Ca granular precipitate Ca3(PO4)2. Bioactivity: presence of Ca in the AH and APH groups, absence in the UT; FE-SEM: removal causes fractures not only at the interface (UT), but also in places of attached bone tissue, mainly in APH.	The surface created with APH obtained well-aligned nanotubes with a dense structure. Precipitates of calcium phosphate and hydroxyapatite were obtained in clusters. Compared to the control, the experimental groups showed significantly higher removal torque.
Li, M. et al., 2022 [67]	-	Mini-pin (original SLA) 5.0 mm × 1.1 mm, n = 144	Immersion in simulated body fluid (SBF) 24 h, 37 °C for BioCaP formation + immersion in SBF 5 × 24 h, 37 °C for deposition of amorphous layer + 20 mL calcium phosphate solution (BSA introduction for SEM reading).	G2—no treatment, bath in PBS + BSA; G3—amorphous treatment; G4—amorphous treatment + BSA; G5—crystalline treatment; G6—crystalline treatment + BSA	G1: no treatment or BSA	3 days, 1, 2 and 4 weeks post-surgery	SEM: analysis of surfaces with and without BSA; FTIR; CLSM; spectrometry; alkaline phosphatase activity and %BIC.	Stability	The thickness of crystalline layers is seven times greater than that of amorphous ones. Treatment by crystalline BioCaP allowed pharmacological transport. There was an increase in bone/Ms contact in the 1st week in G4, but in other groups this increase occurred at 2 and 4 weeks.	The pharmacological transport capacity was 10 times greater in the BioCaP treatment than in the amorphous ones. The contact between bone and Ms increased after the 1st week in the crystalline group, unlike the others, suggesting that this treatment is a technique that can increase stability and increase the success rate of Ms.
Baser, B. et al., 2023 [34]	Polyurethane foam—artificial bone block	Cylindric (BioMaterials Korea, Seoul, Republic of Korea), 8 mm × 1.5 mm, n = 96 (self-tapping and self-drilling)	n = 46 retrieved from patients in previous study + sterilization n = 46 as received from manufacturer All inserted into artificial bone.	n = 46 reused n = 23 self-tapping, n = 23 self-drilling	n = 46 New, n = 23 self-tapping, n = 23 self-drilling		Periotest (mobility test). Torque value tests: maximum insertion. Pull out strength tests.	Primary stability	MITV: used 17.3 Ncm; new 18.9 Ncm; higher values for new self-drilling then for the used. The type of insertion had statistically significant differences. Periotest: only differences in the self-tapping group, *p* = 0.001. Pull-out strength test: 35 of the 46 self-tapping detached from the head, and self-drilling only had plastic deformation.	Used orthodontic MIs showed poor performance compared to unused implants inserted under in vitro conditions.
Byeon, S. et al., 2023 [31]	-	Ti-6Al-4V ELI alloy rods (Fort Wayne Metals Research Products, Fort Wayne, IN, USA)	APH: (Nitric acid (HNO_3_) + HF: H_2_O in the ratio of 12:7:81 for 10 s)+ ultrasonic cleaning in distilled water for 5 min + dryer at 50 °C for 24 h. After, TNT layer was formed: DC eletrostatic device (Inverter tech) 1 h, 20 V, 20 mA/cm^2^ in eletrolyte solution +ultrasonic cleaning + heat treatment. SBF group: immersion in 10 mL of SBF for 5 days and 10 days, at 37 °C. Ibandronate groups: immersion for 10 min or 60 min in ibandronate ar −0.05 MpPa e and lyophilized with N_2_ gas + cleaning H_2_O + repeat for 7 days.	Two groups: AN—immersion in SBF; IB—immersion in ibandronate	UN: untreated, machined	5, 7 and 10 days	FE-SEM	Stability and bioactivity	The nanotubes produced by electrochemically anodizing were fully self-aligned and had a dense structure. Each of the nanotubes had a hollow and independent tube structure with the outer walls attached to adjacent tubes. In the method of continuous immersion of ibandronate for 60 min, the amount of release decreased rapidly. The method of treating six times of 10 min-immersion showed stable release for 7 days.	TNT was formed on the surface of the Ti6Al-4V orthodontic Ms by anodization, and then ibandronate was loaded six times for 10 min to ensure continuous release. It was concluded that this method must be surface treatment to enhance the bioactivity and osseointegration. However, in clinical application, it is necessary to study the biostability and biofunctionality on the surface of Ms according to the concentration of the drug using the sustained release method of the drug, which is the present study.
Gezer, P. et al., 2023 [32]	Polyurethane foam—artificial bone block	TiAl6V4-ELI grade 23 (BioInfinity Orthodontic Screw, Istanbul, Turkiye) (Avrupa Implant, Istanbul, Turkiye) 8 mm × 1.6 mm, n = 36	MAO: Ms as anode and stainless steel plate as the cathode in an aqueous electrolytic bath with phosphoric acid, for 5 min and at 250 V. RBM: calcium phosphate and hydroxyapatite mixture + acid rinse.	n = 12, MAO group: aqueous eletrolytic bath; n = 12, RBM group.	n = 12, no treatment, machined	0 days, 1, 2, 4, 8 and 12 weeks	Torque tests: MIT and MRT.	Stability	Significant differences in MIT were observed between all groups. MRT: only the difference between MS group and RBM-treated group was significant; highest value in the RBM-treated group, lowest MS. A positive significant correlation was found between MIT and MRT in all groups.	RBM-treated group was significantly higher than the Ms group in MIT and MRT values. RBM-treated group was found to be significantly more stable than the MAO-treated group at weeks 8 and 12.
Li, M. et al., 2023 [33]	-	SLA treated Ti plates and smooth SSL plates (diameter: 5 mm, thickness: 1 mm)	Biocap coating: Ti discs were immersed in a biomimetic modified Tyrode (BMT) solution—NaCl: 55.15 mM; CaCl_2_·2H_2_O: 0.94 mM; MgCl_2_·2H_2_O: 0.56 mM; NaHCO3: 1.58 mM; Na_2_HPO_4_·_2_H_2_O: 0.38 mM (4.6 mL BMT per plate) at 37 °C for 24 h. SSL discs immersed for 0, 12, 24, 36 and 48 h, respectively. All were sterilized and immersed in a supersaturated calcium phosphate solution (CPS), namely, NaCl: 10.23 mM; CaCl_2_·2H_2_O: 0.30 mM; Na_2_HPO_4_·2H_2_O: 0.15 mM), buffered with TRIS (3.76 mM; pH 7.4) at 37 °C for 48 h to form a crystalline layer, composed of octacalcium phosphate (OCP).	(2) Ti 24 h BMT CPS group, (4) SSL 0 h SBF BMT group, (5) SSL 12 h BMT CPS group, (6) SSL 24 h BMT CPS group, (7) SSL 36 h BMT CPS group, and (8) SSL 48 h BMT CPS group.	(1) Ti group, (3) SSL group	0, 12, 24 and 48 h	SEM, EDS, surface roughness meter, ELISA, contact angle detection for wettability.	Stability: surface roughness and wettability + surface properties	The morphology, chemical composition and drug loading capacity of the BioCaP coating on smooth SSL were confirmed. This coating improved roughness and wettability of SSL surface. In vitro, with the extension of BMT coating period, the cell seeding efficiency, cell spreading area and cell proliferation on the BioCaP coating were increased.	The BioCaP coating can be applied on the medical grade SSL surface and serve as a carrier system for bioactive agents. The surface properties of medical grade SSL surface such as biocompatibility, osteoconductivity and osteoinductivity by incorporation of osteoinductive agents can be improved by the BioCaP coating.

AE: acid-etching; AFM: atomic force microscopy; AO: oxidative anodizing; BA: bone area; BIC: bone interface contact; BSA: bovine serum albumin; BV/TV: ratio between bone volume and thickness volume; CaP: calcium phosphate; Ca^2+^: calcium ion; CLSM: confocal laser scanning microscope; C1: control group 1; EDX/EDS: X-ray spectrophotometry; ELISA: enzyme linked immunoabsorbent assay; FE-SEM: field-emission scanning electron microscopy; FTIR: Fourier-transform infrared spectroscopy; FTV: fracture torque value; G: group; ISQ: implant stability quotient; HCl: hydrochloric acid; H_2_0: water; ITV: insertion torque value; LED: light emitting diode; LIPUS: low-intensity pulsed ultrasound; LLLT: low-level laser therapy; min—minutes; Ms—miniscrews; MTT: MTT reduction assay for cell viability; NaOCl: sodium hypochlorite; NP1: group without acid-etching 2; PCR: real time chain polymerization reaction; PTV: Periotest value; PTFE: polytetrafluoroethylene; P1: acid-etching group 1; RFA: resonance frequency analysis; RTV: removal torque value; SEM: scanning electron microscopy; SLA: sandblasting, large-grit and acid-etching; Ti: titanium; TiO_2_: titanium oxide; X: not clarified.

**Table 3:** Summary of extrapolated data from included in vivo studies.

Author, Year	Species, Sex and Age	n	MI Type and Number	Surface Treatment (Type, Time, Dose and Protocol)	Experimental Group	Control Group	Follow -Up	Test Used to Evaluate Outcomes	Primary Outcome	Secondary Outcomes	Results	Conclusions
Hassan, A. et al., 2003 [68]	New Zealand rabbit, M, mature	9	Onplant System (Nobel Biocare, Kloten, Suwiter), n = 54	100 µL of rhBMP-2 and 100 µL DMP-1 overnight + 100 µL neutral solution, 5 days	rBMP-2; rDMP-1; B + D	Without PBS immersion treatment	6 s	Description biomechanics: strength assessment. Histological analysis	Stability	Surface properties	Group BMP2 with greater bone formation. Significant bone formation at the O/M interface in groups BMP-2 and B + D; mechanical characterization: BMP-2 with resistance to forces of 3.4–5 kg; B + D 3 kg; DMP-1 and control 0–1.3 kg.	Recommended treatments show potential to improve the osseointegration of implants in immediate orthodontic loading protocols.
Aoki, T, et al., 2005 [72]	Beagle dog, M, 8–10 months	8	PLLA (Fixsorb-MX, China), 8.00 mm × 2.9 mm, n = 16	Poly-L-lactic acid in the constitution and on the surface (PLLA), molecular weight 200,000: bio absorbable material	n = 8, PLLA with load 100 gf and without load, 3 months and 6 months	n = 8, PLLA no charge (0 months)	3 and 6 months	SEM. Tensile strength tests. Histological analysis. Analysis from BA and BIC. Molecular weight measurement	Stability	Surface properties. Tooth movement. Bioactivity	BA was higher in the 3-month group than in the 0-month group (*p* < 0.01); higher BIC in the 3 months than in the control (*p* < 0.001); BA and BIC with no differences between 3 and 6 months. Fracture strength: increased over time, with and without load; greater at 3 months (*p* < 0.001) and at 6 months (*p* < 0.01) than the control; no differences with or without load. Molecular weight: significant decrease at 3 months and at 6 months (*p* < 0.001 in both).	It is suggested that bioabsorbable implants have good biocompatibility and fracture resistance, being a protocol capable of clinical use in orthodontic treatments.
Kim, SH. et al., 2008 [73]	9 M/28 F; 24.53 ± 7.61 years	37	Ti8.5 mm × 1.8 mm (C-implant, Seoul, Republic of Korea), n = 64	Source SLA	G1: delayed load (0–3, 3–6, 6+ months)	G2: Immediate load	0–22 months	Digital measurement of removal torque	Stability		Average RTV of 16.4 N/cm. No influence of age, malocclusion and load. From 6 months: RTVs significantly higher, 23.7 N (unloaded) vs. 15.1 N (0–3 months) vs. 20.7 N (3–6 month).	The longer treatment time, the greater the RTV. Treatment offers stability and resistance, without difficulty in removal.
Kim, TW. et al., 2008 [74]	Beagle dog, M and F, 20 and 14 months	Two (one M and one F)	Jeil (Jeil Medical, Republic of Korea), 6.0 mm × 1.6 m; n = 2 × 40 = 80	n = 40 10 µm microgrooves on the external surface	n = 40 (2 in buccal, 8 in palatal/lingual in the right maxilla and mandible)	n = 40; no treatment	16 s	Success rate analysis; BIC and BA analysis	Stability	Bone area of pressure and tension	BIC: no differences between groups, except for the pressure site, which was greater in the group with microgrooves (*p* < 0.01); higher pressure sites in the control and lower than tension sites (*p* < 0.01), with no differences in the experimental group. Histology: samples with osteointegration present: Haversian systems, lamellar and new bone.	The Ms with microgrooves had a perpendicular arrangement of the connective fibers, while the control had parallel ones. Microgrooves can have effects on the way the connective gingival tissue is positioned, which can positively affect bone adaptation.
Chang, CS. et al., 2009 [75]	New Zealand White rabbit, adult	24	AbsoAnch (DENTOS Inc., Daegu, Republic of Korea), 1.3 mm diameter, n = 144	SLA: Al_2_O_3_ particles, 355–425 µm, 4 kg/cm^3^+ AE HCl/H_2_SO_4_ 70%, 80 °C, 30 min. SL/NaOH: SL+ immersion 5 M NaOH 60 °C, 24 h + cleaning + drying + heating at 600 °C in a furnace	n = 48, SLA; n = 48 SL/NaOH	n = 48, no treatment	2, 4, 8 and 12 s	Digital measurement of removal torque. FE-SEM: %BIC	Stability	Surface properties	RTVs: higher in SLA and SL/NaOH. SEM: SLA with microscopic roughness at two levels. SL/NaOH with macroscopic roughness. RTVs: SLA increased significantly after 4 weeks; SL/NaOH increased after 8 weeks and at 12 weeks there were different (higher) SLA values (*p* < 0.05); after 12 weeks, %BIC was higher in the experimental groups than in the control group. There is a correlation between the %BIC and RTVs.	SLA and SL/NaOH treatments can increase removal torque under orthodontic loading. SLA allowed faster improvements in RTVs at 4 weeks, while SL/NaOH achieved increases only after 8 and 12 weeks. The expression of %BIC was different than that of RTVs, with a correlation between the two.
Kim, SH. et al., 2009 [13]	Dog, M, 7–11 months	12	8.5 mm × 1.8 mm (C-implant, Seoul, Republic of Korea), n = 96	SLA	n = 48, SLA	n = 48, Without treatment	3 and 8 s	Success rate. Measurement of torque values: analysis of total absorbed energy (TIEV and TREV)	Stability	-	Success rate 50% (33 lost): Control 52.1% and SLA 47.9%. MITV and MA of the SLA group were significantly lower than control (*p* = 0.034 and *p* = 0.039). TREV significantly higher in SLA than control (*p* = 0.046). No differences between SLA and control in TIEV, MRTV and RAMV. No differences between clockwise or counterclockwise rotation.	Ms treated by SLA have lower values of torque and angular momentum at insertion, but higher values of total energy absorbed than the control. The osseointegration created by the treatment, and influenced by the location and rotation, is sufficient to resist orthodontic forces.
Niwa, K.et al., 2009 [76]	White Japanese rabbit	21	Central hook in Ti that connect Ms to a 5 mm disc in pure perforated Ti; n = 6 × 21= 126	1. Plasma spray method—flame heat β-TCP powder, depositing α-TCP on the surface of the disc; 2. Hydrothermal processing of α-TCP	n = 42 T1: α-TCP; n = 42 T2: HA	n = 42, no treatment	1, 2 and 3 s	Radiographic evaluation: Ms position and bone characteristics; tensile strength test. Histological analysis.	Stability time	Deposition of compounds. Surface morphology. Healing	Bone contact: notable radiopacity in α-TCP and HA (2 weeks). Bond strength: extensive bone formation in all groups: the α-TCP group showed a larger area than the AH group (*p* < 0.01) and both larger than control (*p* < 0.01). Average elastic strength in Mpa: 1 week—C 0.006, T1 0.003, T2 0.019; 2 weeks: C 0.365, T1 1.004, T2 1.063; 3 weeks: C 2.849, T1 3.686, T2 3.161.	Two weeks after placement: formation of new osteoid tissue around Ms treated with α-TCP and HA. At 2 weeks, greater bone volume was formed in the disc perforations of the α-TCP group than in the HA.
Mo, S.S. et al., 2010 [77]	White rabbit	42	9.5 mm × 1.8 mm (C-implant, Seoul, Republic of Korea), n = 412	SLA	N = 180, SLA	n = 160, no treatment	2 days, 1, 6 and 10 s	Digital measurement of removal torques. Measuring success rate	Stability	-	Failure of 13 Ms. Success rate: 96.8%. RTVs: higher values for the SAE group (*p* < 0.0001); significant difference between load periods (*p* < 0.001); no differences between loaded or unloaded groups.	SLA treatment for Ms, subject to different loading protocols, allows similar success rates between them. RTVs were higher for SLA treated group than machined control during the first 6 weeks of healing.
Cho, I.C. et al., 2012 [78]	Beagle dog, M, 1 year old	6	Ti-6AL4V (BioMaterials Korea, Seoul, Republic of Korea), 8 mm × 1.45 mm, n = 54 = 18+ 36	(n = 18) SLA: 100 µm aluminum particles, 2 min, 2 bar + 30% HCl, 60% H_2_SO_4_ and diluting solution. (n = 18) SLAO: SLA + oxidative anodization 3 min, 250 V	n = 6 + 12SLA; n = 6 + 12 SLAO	n = 6 + 12, no treatment	8 s	Digital measurement of insertion and removal torque: analysis of maximum torque, total energy and peak energy	Stability	-	Three highest insertion values in control (*p* < 0.01); removal: no differences in VTE and NPE between groups, with SLAO having higher MRTV than SLA and control (*p* < 0.001).	SLAO treatment can be an effective way to reduce the damage due to the insertion of Ms, both in the tissue and in the device, and can also improve its stability.
Choi, S-H. et al., 2012 [79]	Beagle dog, 1 year old	2	7 mm × 1.5 mm (BioMaterials Korea, Seoul, Republic of Korea), n = 8	AO + immediate load	AO	No treatment	12 s	Measurement of initial torque and mobility. SEM and AFM: surface and roughness analysis	Stability	Surface properties	No differences in torque and initial mobility between groups. SEM: used AO group tip turns become smooth. AFM: roughness of the turns of the AO group used significantly lower than in its initial state (*p* < 0.05); turns of group AO used rougher than those of group C used and not used (*p* < 0.05).	Ms subjected to AO obtained better surface characteristics than machined surfaces, despite differences in texture at insertion and in the initial loading period.
Karmarker, S. et al., 2012 [80]	White rabbit, 10 months	3	Absoanch (DENTOS Inc., Daegu, Republic of Korea), 6 mm × 1.3 mm, n = 36	n = 18, AO	n = 18, anodized surface	n = 18, no treatment	6 s	Digital tests, maximum insertion and removal torque	Stability	-	ITVs: no differences between groups. RTVs: higher in the anodized group than in the control (3.79 ± 1.39 Ncm vs. 2.05 ± 1.07 Ncm) (*p* < 0.01). The interface forces calculated from the RTV were 10.6 MPa and 5.74 MPa for the experimental and control groups, respectively.	Anodizing of Ms can enhance initial stability, through greater retention capacity, enhancing absolute anchorage.
Omasa, S. et al., 2012 [81]	Sprague-Dawley rat, M, 6 weeks; Fischer mouse, M, 6 weeks	30; 9	Ti (MogiShokai CO, Japan), 7.3 mm × 1.4 mm (Keisei Medical Industrial CO, Tokyo, Japan) 3.5 mm × 1.5 mm; n = 78	LLLT (Ga-Al-Ar) at 830 nm, 200 mW, 195 J/cm^2^,135 s × 2 points, 54 J/session, 1×/day, 7 days	GLLLT, n = 39; LLLT, right tibia	n = 39, no treatment, left tibia	7 and 35 days	Micro-CT: levels of new bone formation; PCR: BMP-2 expression analysis	Stability and osteogenesis	-	Osteogenesis: new bone formation observed at 5 days in both groups, but stronger in LLLT; BV significantly higher in the LLLT group than in the control on day 7 (1.53× higher); BMP-2 expression: 1.92× higher in LLLT than in the control on the 1st day, but similar in the rest.	LLLT therapy improved the stability of Ms and accelerated bone formation around them by increasing BMP-2 gene expression.
Uysal, T. et al., 2012 [82]	New Zealand White rabbit, M, adults	15	Ti Dual-Top 8 (Jeil Medical, Republic of Korea) mm × 1.4 mm, n = 60	LED: Osseo Pulse LED (Biolux Research), 618 nm, 20 mW/cm^2^, 20 min, for 10 days	n = 10 × 3 LED (G1 0 cN, G3 150 cN and G5 300 cN)	n = 10 × 3 No treatment (G2 0 cN, G4 150 cN and G6 300 cN)	1 and 21 days	RFA (OsstellMentor): stability assessment	Stability	-	Similar initial stability among all groups. Significant differences in the ISQ quotient between the experimental group and the control; significant increase between groups of forces in the LED group; in control, the greater the force, the lower the ISQ.	Photobiomodulation therapy with LED light achieved greater stability than control over the 21 days, and may have a favorable effect on healing and insertion of orthodontic Ms.
Cho, Y.C. et al., 2013 [83]	Beagle dog	4	Model 1016106 (Orlus™, Ortholution, Seoul, Republic of Korea) Original SLA 1.45 mm × 6 mm, n = 32	Laser beam with plasma ion penetration at low temperatures	n = 16, treatment plasma ion implantation; source SLA	n = 16, without treatment; origin SLA	0–12 s	Insertion torque sensor; periotest: mobility; histological analysis	Stability	Mobility, bone parameters, success rate	SR: 100% for control; 93.75% experimental group. Insertion torque: slightly higher experimental (*p* = 0.61). Contact: control 64.2% and experimental 72.1%, 3 weeks; 12 weeks, control 66.2% and experimental 63.4%. (*p* = 0.11 and *p* = 0.65).	Ion-modified Ms have similar biological characteristics to the control in terms of the outcomes evaluated.
Pinto, M. et al., 2013 [84]	New Zealand rabbit, M, 4 months	16	Ti_6_Al_4_V: INP and TF 9 mm × 1.5 mm, n = 32 × 2	LLLT: 21 days, 2 days apart, 10 sessions, external and internal point of the right tibia, 90 J/cm^2^, 25 s, 2.5 J.	n = 32, LLLT in both brands, right tibia	n = 32, without treatment of both brands, left tibia	21 and 36 days	Mechanical removal test: measurement of load, maximum force and displacement values	Stability	-	Control: (vs. TF) 108.58 ± 17.92 to (vs. IND) 124.63 ± 13.43 N/cm². LLLT: 137.37 ± 11.78 (IND) to 177.39 ± 24.9 (TF) N/cm². Greater removal force for treated groups. Significant difference between TF control and INP control (*p* < 0.05). Values increased in groups with LLLT, especially in the TF group.	Low-intensity laser therapy was able to increase the stability of Ms. All types of Ms observed are effective for clinical use, with or without LLLT.
Cuairán, C. et al., 2014 [85]	Dog without breed, M, 1–2 years	3	Ti_6_Al_4_V(Neodent, Curitiba, Brazil), 5 mm × 1.6 mm, n = 60	Treatment with Zoledronate: 16 µg in 50 µL of phosphate saline solution at the site of the burr cavity, before insertion	GZ: n = 30, Zoledronate Injection	n = 30, no treatment (injection of 50 µL of saline solution)	0–8 s	RFA (Osstell Mentor): stability assessment; micro-CT: BIC 3D	Stability longitudinal	Scarring around the MI	Stability: significantly less stable controls (*p* < 0.05). BIC: superficial bone layers with less bone in both groups than the two deeper layers (*p* < 0.05); at 8 weeks, greater amount of cortical bone around the control and greater amount of bone trabecular in GZ.	A single, localized dose of Zoledronate was able to prevent significant loss of stability over time, with control demonstrating significant losses at 4 weeks and an increase in the 6th, losing again until the 8th week.
Miura, K.et al., 2014 [86]	Sprague-Dawley rat, M, 6 months	7	Ti 1.4 mm × 4 μm; n = 14	LIPUS, 15 min/day, 2 weeks; I:30 mW/cm^2^, F:3.0 MHz at 20% cycle;	LIPUS + right tibia implant	No treatment + implant left tibia	2 s	FE-SEM: O/M contact ratio	Stability	Mobility (periotest)	Experimental group: good O/M contact; O/M Ratio = 72.9 ± 10.2%. Control: gaps in the O/M interface; O/M Ratio = 52.3 ± 9.0% (*p* < 0.05)	LIPUS increased bone/microimplant contact and reduced microimplant mobility in growing rats.
Oh, EJ. et al., 2014 [44]	Wistar rat, M, 7 weeks	16	Ti_6_Al_4_V (Jeil Medical, Republic of Korea), 4 mm × 1.4 mm; n = 32	n = 16 APH treatment: Glycerol/H_2_O/NH_4_F solution, 20 V, 1 h + cyclic calcification by immersion in NaH_2_PO_4_ (0.05 M), 80 °C and Ca(OH)_2_ (100 °C) 1 min/cycle for 30 cycles + heat treatment 500 °C, 2 h	(n = 16)APH; AH:anodizing + heat	(n = 16) UT: no treatment	3 days, 3 and 6 s	FE-SEM; roughness tests (Surftest SV-3000); microscopy: hydrophilicity; digital torque sensor; EDS/XRD; histological analysis: %BIC	Stability	Surface properties	The smaller the contact angle, the greater the roughness; higher in APH. Biomechanical stability/strength: higher RTVs in APH at 3 and 6 weeks (*p* < 0.05); RTVs: higher at 3 and 6 weeks (*p* < 0.05). Osseointegration: little bone formation at 3 s in UT, unlike APH (%BIC: UT8.25 ± 6.67% vs. 84.00 ± 8.47%, *p*< 0.05); at 6 weeks formation occurred better and faster in APH (*p* < 0.05): better stability.	APH treatment accelerated the formation of hydroxyapatite, improved bone formation and presented surfaces with bioactivity and biocompatibility, which will allow an improvement in the initial stability of Ms. Indication for poor quality bone, where rapid healing and osseointegration is required.
Oh, NH. et al., 2014 [87]	New Zealand White rabbit	12 (n = 6 diabetic and n = 6 healthy)	8.5 mm × 1.8 mm (C-implant, Seoul, Republic of Korea); n = 48	SLA. Experimental group in diabetic rabbits induced for 4 weeks	Diabetic rabbits, treated with SLA and without treatment	Healthy rabbits, treated with SLA and without treatment	4 and 8 s	Body mass measurement; blood glucose test; insertion and removal torque and energy tests; %BIC Analysis	Stability	Body mass and blood glucose levels	ITVs: no significant differences between groups and treatments, but higher total insertion energy in the control. RTVs: no differences between diabetics and non-diabetics, but with higher total removal energy in the diabetic group, without significant difference (*p* > 0.05); in the diabetic group, slightly higher removal torque and energy in the SLA group, but control without differences in treatments. %BIC: higher in control than in diabetic patients, with no statistical difference between the two.	The use of Ms in diabetic patients will have similar results as in healthy patients. Surface treatments in these conditions also seem to be applicable to diabetic patients, with results consistent with those obtained for healthy patients.
Yadav, S. et al., 2015 [50]	White New Zealand rabbit, M, 4–5 months	8	6 mm × 1.6 mm n = 128	G2 (n = 32): AE: 0.11 mol/L and HCl for 20 min; G3 (n = 32): gritblasting with alumina; G4 (n = 32): gritblasting+ AE 0.11 mol/L HCl 65 °C for 20 min	G2: AE (n = 32); G3: gritblasting (n = 32); G4: gritblasting+ AE (n = 32)	n = 32, G1: no treatment	8 s	Profilometer Goniometer. Removal torque test; histological analysis: BIC	Biological integration	Stability	RTVs: no significant interaction between bone type and surface. Medium roughness: Ra—G4: 3.69; G3: 4.88; G2: 1.78; G1 1.13 *p* = 0.001. Rq—G4 4.87; G3 7.06; G2 3.27; G1 2.56 *p* = 0.001. O/M Contact: significantly greater in G4 than G3, G2 and G1 and with no differences between them. Average %BIC: Tibias—G4 66.34%; G3 53.07%; G2 50.64%; G1 39.30%; Femur—G4 68.94%; G3 49.10%; G2 48.30%; G1 45.28%.	Contact with liquids was greater for the control and lower for gritblasted with conditioning. The removal torque in both the tibia and femur was greater in the gritblasted with conditioning group.
El-Wassefy, N. et al., 2015 [88]	White New Zealand rabbit, M, 6 months	4	Absoanch (DENTOS Inc., Daegu, Republic of Korea) 7 mm × 1.4 mm, n = 40	G1: autoclave 30 min at 121 °C and 18 psi; G2: gamma ray sterilization for one night at 25 kGy; G3: UV sterilization, 90 min 254 nm	n = 30; G1: autoclave; G2: gamma rays; G3: UV light	n = 10, unused, untreated	30 days	SEM; absorption spectrometry; healing analysis; analysis bone histology	Property surface and mechanics (potential reuse)	Ionic release; stability	All Ms had stability, good mechanical fixation and no signs of inflammation or reaction.	Autoclave sterilization treatment showed better results in terms of cellular activity than the other treatments.
Ganzorig, K. et al., 2015 [47]	Sprague-Dawley rat, 6 weeks	40	T_i6_Al_4_V (Nishimura Metal, Japan) and 1.6 mm × 1.5 mm SS, n = 140	LIPUS: Osteotron_D IB, Ito Co; 1.5 MHz, 30 mW/cm^2^; 24 h after insertion, 20 min/day in the right tibia (1×/15 min in cells)	GLIPUS, n = 80, LIPUS, right tibia	n = 80, without treatment and left tibia	0–14 days	SEM and micro-CT: Bone morphology, CBT (mm and %) and BIC	Stability and osteogenesis and	Morphology surface and cellular	From day 3 to day 14, BIC gradually increased in all groups. LIPUS increased BD density, CBT and bone formation rate after insertion (*p* < 0.05). Increase in GLIPUS of ALP regulation in vitro, on the 3rd day (*p* < 0.05).	It is suggested that the application of LIPUS improves bone formation around titanium alloy and stainless steel Ms and may therefore improve their initial stability and success rate throughout the treatment.
Goymen, M. et al., 2015 [89]	New Zealand White rabbit, M, 6 months	17	Ti6Al4V (Jeil Medical, Republic of Korea), 8 mm × 1.4 mm, n = 68	n = 48 LLLT: GaAlAs diode laser (Cheese dental laser, Wuhan Gigaa Optronics Technology Co. Ltd.), 810 nm, 0.3 W, area 5.85 cm^2^; 195–390 s/point for 10 days.	G2 (n = 12):LLLT 10 J/cm^2^ without load; G3 (n = 12): LLLT 20 J/cm^2^ without load; G5 (n = 12): LLLT 10 J/cm^2^ with 150 g load; G6 (n = 12): LLLT 20 J/cm^2^ with load 150 g	G1 (n = 8): without LLT and without load; G4 (n = 12): without LLT and 150 g load	10 days and 4 s	Digital measurement of initial torque %BIC and BT analysis	Stability	-	BIC: G6 with the highest value of 83.11 ± 1.75 (*p* < 0.05), followed by groups 5 and 3, group 1 with the lowest value of 36.15 ± 2.45; presence of differences between all groups. BT: significant differences between group 1 and each of the others (*p* < 0.05); group 1 with the lowest value and group 3 with the highest (1.93 ± 0.31 compared to 2.16 ± 0.16), followed by 4 and 2; no significant differences between groups. No significant correlation between BIC and BT values.	Using Ms as orthodontic anchorage is easy, effective and a reliable method. The BIC values of the groups with 20 J/cm^2^ (3 and 6) were higher than the others. LLLT can be a method that allows increasing the stability of M. There was no relationship between BIC and BT values.
Jang, I. et al. 2015 [90]	White New Zealand rabbit, F, 13–14 weeks	4	Dual Top 6 mm × 1.6 mm (Jeil Medical, Republic of Korea), n = 8	n = 4 AO in 2 steps: Electrolyte bath. Drugs inside nanotubes.	n = 4 G2:TiO_2_ nanotube array	n = 4 G1 without treatment	8 s	FE-SEM: morphological characteristics of Micro-CT microimplants: %BIC and %BV/TV	Stability	Tissue and bone volume	Experimental groups with higher average Ms/bone contact than control (52.8 ± 18.1% vs. 29.3 ± 15.6%, *p* = 0.016). Tissue and bone volumes were also higher in the experimental group (BV/TV%): AO 81.2 ± 4.2% and C 73.3 ± 16.4%, *p* = 0.386.	Nanotubes promoted a rough surface and greater contact with bone, allowing osseointegration. Physical properties were maintained after use in rabbits.
Liang, Y. et al., 2015 [48]	Sprague-Dawley rat, F, 3 months	36 (excised number of ovaries n = 24)	6.6 mm × 1.5 mm, n = 72	Application of strontium (Sr): polishing + HCl + CaCl_2_ + ultrasonic cleaning in acetone, 10 min + H_2_O + drying + ECD (electrochemical deposition with platinum mesh)	n = 12 G1: Without ECD, ovariectomy; n = 12 G2: ECD with Sr, ovariectomy	n = 12, no treatment + without ovariectomy	2 and 4 s	Histological analysis. Confocal microscopy: determination of DDL, MAR and MS/BS. Measurement maximum removal torque	Stability	Success rate. Surface properties	Histological analysis: G1—less bone formation and poor continuity; G2 new bone formation and increased lamellar layer thickness and discontinuity. %BIC, BV%TV and BT significantly higher in G2. RTVs: G2: 27.94 ± 1.43, control 22.04 ± 2.11 and G1 25.30 ± 1.38 N.cm, with significant differences between experimental and control (*p* < 0.001) and between experimental (*p* < 0.01).	Strontium treatment is easily performed by ECD. This has a promoting effect on the osteointegration of Ms in animals with osteopenia and may be a new treatment protocol for patients with osteoporosis.
Miyawaki, S. et al., 2015 [45]	White New Zealand rabbit, 1 M, 1 F, 14 weeks	2	Ti 6 mm × 1.6 mm, n = 4	SpikeAnchor, Ti_6_Al_4_V, two portions: portion that receives forces and portion with spikes that contact the cortical bone	Implantation in the femur with SpikeAnchor	No Spike	4 s	Lateral displacement test: mechanical retention forces; visual analysis of device insertion	Stability	Depth implementation	Retention force was significantly greater in the experimental group at different displacements (*p* < 0.05). The smaller the displacement, the greater differences in the forces applied in the experiment. The spikes were implanted 0.3 mm after application of compressive forces.	SpikeAnchor allowed automatic implantation over time of spikes into cortical bone. There was an increase in Ms stability of 3–5 times when compared to the experimental group and the control, with absolute anchorage.
Sirisa-Ard, A. et al., 2015 [91]	New Zealand rabbit, M, adults	24	Ti_6_Al_4_V (Russell Symes & Co, Australia), 6 mm × 1.5 mm, n = 47	n = 23, SLA treatment	n = 23, treatment SLA	n = 24, without treatment (MA)	8 and 16 s	Digital removal torque tests; %BIC analysis	Stability	-	RTVs: at 0 s, SLA group with higher values than MA (7.21 Ncm vs. 5.38 Ncm, *p* < 0.05); at 8 s, MA group with higher values than SLA (8 Ncm vs. 6.59 Ncm), but without significance; comparing the 0 s and the 8 s, there were no significant differences in the SLA group. %BIC: statistically significant decrease in values from 0–8 weeks for MA (*p* = 0.003); higher in the SLA group at 8 s.	SLA surface treatment does not increase the removal torque of Ms.
Tabuchi, M. et al., 2015 [49]	Sprague-Dawley rats, 8 weeks	6	6 mm × 1.4 mm (Jeil Medical, Republic of Korea), (n = 12)	UV light for 12 min with TheraBeam Super Osseo device.	UV Light	No treatment	3 s	SEM and EDX; ELISA; CLSM; mechanical Tests at 0.5, 10, 15, 20, 25, 30, 35 and 40 N	Stability	Surface characteristic; cell adhesion, behavior and proliferation	Lateral pressure test: smaller movement in the photofunctionalized group (*p* < 0.05, except at 40 N); the greater the force, the greater the displacement, but photofunctionalization reduced this effect, except at 40 N (*p* < 0.01); at 3 weeks experimental group: continuous bone, newly formed, without visible interface between the cortex and the space of the marrow.	The displacement of photofunctionalized Ms was 30–40% less than untreated ones. It is suggested that photofunctionalization increases the bioactivity of the Ms under study, as well as their anchoring capacity and stability, without changing the mechanical morphology itself.
Tabuchi, M. et al., 2015 [92]	Sprague-Dawley rat, M, 12 weeks	-	Ti_6_Al_4_V (Jeil Medical, Republic of Korea), 6 mm × 1.4 mm	Ultraviolet light: TheraBeam Super Osseo, 12 min	UV Light	No treatment	3 s	SEM. Measurement of insertion and removal torque. Lateral force resistance analysis. EDX.	Stability	Surface properties	Initial SEM: control with hydrophobic surface; UV became superhydrophilic. RTVs: no differences at 0 weeks; at 3 weeks, higher in UV than control. Final SEM: UV group with regenerated bone more intact and with continuity than control. Faulty, non-cohesive O/M interface in both groups. Resistance to lateral force: lower in the control group, with a displacement 1.4× greater than UV.	The surface treated by UV converted from hydrophobic to superhydrophilic. At 3 weeks of healing, the removal torque in the UV group was greater than control. The interface between complexes was faulty, with non-cohesive fracture. Subject to lateral forces, the displacement was greater for the untreated group.
Villani, G. et al., 2015 [93]	Dog without breed, M, adult	6	Ti_6_Al_4_V (Conexão, São Paulo, Brazil), 6.0 mm × 2 mm, n = 36 = 6 × 6	AE: HNO_3_ + HCl + H_2_SO_4_.	n = 18, AE (rough R), with and without load 1.0 N	n = 18, Untreated (smooth S), with and without load 1.0 N	16 s	Digital measurement of insertion and removal torques. Periotest. Digital displacement measurement.	Stability	-	No significant performance differences between groups. ITVs high and low initial mobility in all groups. RTVs smaller than ITVs in both groups. AE allowed a rougher surface, which resulted in higher RTVs and lower mobility than control, and therefore, greater secondary stability, but *p* > 0.05.	Higher success rate for Ms treated with acid-etching. Primary stability greater than stability after 16 weeks. No differences in stability between groups, according to the parameters evaluated.
Bayani, S. et al., 2016 [94]	German Shepherd dog M, 6–8 months	3	8 mm × 1.6 mm (Jeil Medical, Republic of Korea), n = 60	n = 30 Treatment with growth factor obtained from plasma (PRGF)	n = 30, PRGF, sub-groups with (150 g) or without immediate load	n = 30; without treatment, sub-groups with or without immediate loading	12 s	Maximum removal torque measurement test	Stability primary	-	RTVs: there were differences between the four groups—GPRGF without load > GPRGF with load > Gcontrol without load > Gcontrol with load.	Application of PRGF significantly increased the removal torque and, therefore, the stability of the Ms.
Cha, BK. et al., 2016 [95]	New Zealand White rabbit, 13–14 weeks	5	Dual-Top (Jeil Medical, Republic of Korea)), Ti_6_Al_4_V, 6 mm × 1.6 mm, n = 12	TiO_2_ nanotubes by AO + machined tunnel (1.5 mg/mL rhBMP-2 transport system): anodization with glycol-ethylene and 0.5 wt% NH4F at 60 V 60 min+ ultrasonic cleaning; anodizing again after opening the window on the nanotubes 15 V, 15 min + tunnel	GTM: n = 4 machined tunnel; GTNM: n = 4 TiO_2_ nanotubes + tunnel	GCM n = 4, no treatment	8 s	micro-CT: bone assessment of %BV and %BS. Microscopy %BIC, %BA	Stability	Surface properties	%BIC and %BS: similar GTM and GTNM values, but higher than the control, but without significant differences. %BA: new bone formation at 3 and 6 weeks in tunnel groups, and this is greater than control. No beneficial effect of nanotubes added to the tunnel was found.	Ms with machined tunnels can be coupled to TiO_2_ nanotube arrays. Pharmacological transport of rhBMP-2 to bone was successful. Bone surface values are higher in the experimental groups (nanotubes > tunnel only), as well as the amount of new bone formation. It would improve osteogenesis performance, but it has not been proven that nanotubes have additional effects to those of tunnel itself.
Choi, S-H. et al., 2016 [96]	Beagle dog, M, 12–15 months	12	Ti_6_Al_4_V (BioMaterials Korea, Seoul, Republic of Korea), 7 mm × 1.45 mm, n = 96 = 8 × 1 2	n = 48, AO: immersion in solution with electrolytes for 3 min, at 250 V	n = 48 AO	n = 48, no treatment	3, 9 and 12 s	SEM, AFM, insertion and removal torques, analysis of %BIC and %BV/TV	Stability	Surface properties; success rate	ITVs (N.cm): similar in both the 3-week and 12-week load groups. RTVs (N.cm): no significant differences between groups at 12 weeks. BIC and BV/TV: decrease in values, but without significant differences.	The maximum insertion and removal torque values, as well as BIC and BV/TV ratios after 3 and 12 weeks, of the anodized group, are not significantly different. The treatment has not proven to be clinically superior.
Espinar-Escalona, E. et al., 2016 [28]	New Zealand White rabbit	10	MI Ti pure (HDC Company, Sarcedo, Italy), 9 mm × 2 mmn = 20	AE: immersion in 0.35 M HF, 15 s, 25 °C. Gritblasting: alumina particles 600 µm, 0.25 MPa until roughness saturation. GBA: acetone, 15 min + H_2_0 + drying in N_2_	n = 5 AE: condition then acidic; n = 5 GB—alumina beam; n = 5 GBA—AE + GB	n = 5 No treatment	10 s	FE-SEM: O/M interface analysis. Manual measurement of removal torque. %BIC analysis.	Stability	Surface properties	Roughness of GB and GBA significantly greater than control and AE, with AE greater than control. %BIC:GBA 79 ± 12% and GB 75 ± 15% (*p* < 0.05), AE 26 ± 6%, C 19 ± 7%. No differences in removal torque between AE and control groups, but higher values for GB and GBA. Greater roughness and wettability in treatments than in control, leading to higher %BIC and higher RTV.	Surface treatments on Ms such as AE, GB and GBA are effective in modifying the surface and improving osseointegration and stability of the device. These create removal torques that do not compromise stability and do not promote fractures. Wettability was the parameter that showed the most influence on torque, based on the values obtained in AE.
Gansukh, O. et al., 2016 [97]	New Zealand rabbit, 3 months	24	Dual-Top ((Jeil Medical, Republic of Korea)), Ti_6_Al_4_V, 6 mm × 1.6 mm, n = 96	RBM (resorbable blasting media) treatment: sandblasting with RBM, CaP and HNO_3_	n = 48, RBM	n = 48, no treatment	0, 2 and 4 s	SEM; measurement of maximum insertion and removal torques; RAM; analysis of %BIC and %BA	Stability	surface properties	SEM: rough and reticulated RBM surfaces; smooth control; higher roughness values in RBM. Mechanical analysis: RBM group with lower MIT values and higher MRT and RAM than the control (*p* < 0.05). No difference in BIC at 4 weeks, but higher BA in the RBM group. Control presents new bone formation after resorption.	RBM surface treatment can maintain the initial stability of Ms.
Kang, HK et al., 2016 [51]	Beagle dog, F, 10–15 months	6	316 stainless steel, 6 mm × 1.2–1.3 mm, n = 48	Nd-YAG 1064 nm Q-switched	n = 12,Nd-YAG Q-Laser switched	n = 12, without treatment	0 and 8 s	Survival rate; insertion torque; micro-CT; histological analysis; mobility	Stability	Surface properties: roughness, topography	No failures. Surface roughness: higher values in the treated group (*p* < 0.05), triple increase; no differences in RTVs and 2D and 3D BIC between groups. There was no increase in fracture resistance, despite an increase in surface roughness.	Surface roughness increased by more than three times due to surface reactions created by the laser. No differences in secondary stability between treated and control groups.
Kim, H.Y. et al., 2016 [52]	White New Zealand rabbit, M,	25	OSSH1606 (Osstem Implant, Seul, Republic of Korea), 1.6 mm × 6 mm Ti6Al4V, n = 150	G1: hydrochloric and nitric AE; G2: RBM, Ca_3_(PO_4_)_2_ beam and acid wash; G3: Ca_3_(PO_4_)_2_ and acid wash	G1, G2 and G3	G0, no treatment	1, 2, 4 and 8 s	Torque test, SR; histological analysis; SEM; EDS	Stability	Sharpness	Osseointegration: at 4 weeks, control RTV significantly decreased, but was increased in G1 (*p* < 0.05 in both); G4 with increased values at 2 weeks, and with higher values at 8 weeks (*p* < 0.05). Calcium and phosphorus infiltration on the bone-like surface was detected in the hybrid/G4 group.	Partial/hybrid RBM group with greater stability compared to the other groups, without a reduction in cutting capacity.
Takahashi, M. et al., 2016 [98]	Sprague-Dawley rat, M, 6 weeks	12	Pure Ti, 4 mm × 1.4 mm; n = 24	n = 12, UV Light, 15 min, on the TheraBeam Affiny device, Ushio. Load and no load immediately	n = 12 ultraviolet light. Insertion into the right tibia	n = 12; no treatment. Insertion into the left tibia	2 s	Micro-CT: bone assessment of BT and BD; FAITH-SEM:O/M contact analysis (BSC)	Stability	Density and bone thickness	FE-SEM: controls showed some gaps at the O/M interface; UV groups with uniform contact; with loading or without immediate loading, BSCs were higher in the UV groups than in the controls (UV 72.7 ± 9.9% and 71.5 ± 16.5% vs. 38.1 ± 26.8% and 40.4 ± 23.5%, *p* < 0. 05).	In this way, UV light on Ms subject, or not, to immediate loading, improved their stability by increasing their contact with the underlying bone.
Jang, I. et al., 2017 [99]	White New Zealand rabbit, X1 3–14 weeks	6	Dual Top 6 mm × 1.6 mm (Jeil Medical, Republic of Korea), n = 24	n = 18 TO 2 steps, in an electrolyte bath. Drugs inside nanotubes	n = 18 = 3 × 6 G2: OA without drug; G3: OA with RhBMP-2; G4: AO with ibuprofen	n = 6 G1 without treatment	8 s and 5 days	Method for detecting drug transport. Histological analysis: % BIC	Stability	Pharmacological transport capacity	Ibuprofen group with significantly higher BIC than control (71.6% vs. 44.3%). Successful use of nanotubes as a carrier. %BIC: Control: 44.3 ± 23.7% G2: 60.1 ± 13.7% G3: 24.6 ± 20.7% G4: 71.6 ± 17.8% *p* < 0.05.	Ibuprofen acted as an anti-inflammatory, allowing better osteointegration than in other groups.
Fernandes, DJ et al., 2017 [20]	New Zealand White rabbit, F, 6 months	12	Ti_6_Al_4_V grade V (ASTM, West Conshohocken, PA, USA) 6 mm × 1.5 mm, n = 48	AE: (HNO_3_ + H_2_O + H_2_SO_4_) under magnetic stirring + HNO_3_ again	n = 24 AE (1, 4 and 8 s)	n = 24, no treatment (1, 4 and 8 s)	1, 4 and 8 s	AAS: analysis of blood concentration of Al and V ions. Measurement of insertion and removal torque. Histological analysis	Stability	Surface properties. Blood concentrations and histological analysis	Surface: intercommunicating micropores in the experimental group; larger TiO_2_ layer in treated Ms, and with lower % of Al and V. Torque: higher values for treated group and ITVs > RTVs. Histological analysis: dense Ca/P particles with proliferating osteoblasTS at the O/M interface of the treated group at 4 s, new bone formed at 8 s.	Acid-etching treatment of the surface of Ms improves surface morphology and mechanical stability, with early signs of osseointegration. It also allowed a reduction in the release of Al and V ions.
Maino, B. et al., 2017 [100]	New Zealand White rabbit, M, 6 months	8	Ti_6_Al_4_V Spider Screw (HDC Company, Sarcedo, Italy), 6.5 mm × 1.5 mm, n = 64	SLA	n = 32, SLA, with and without load 100 g	n = 32, without treatment with and without load 100 g	12 s	X-ray. Digital measurement of removal torque SEM. Histological analysis	Stability	Surface properties	SEM: visible bone adhesion to SLA Ms. RTVs: significantly higher for SLA than control, with and without load. Histological analysis: loaded group, presence of irregular fibers, with modified cortical bone around, indicating remodeling and bone loss. %BIC: no significant differences. Decreased cortical bone area significantly in SLA under load.	Grade V titanium in SLA Ms has greater bone retention than machined surfaces, both at 0 and 3 months. This is due to better interconnection with the tissues. The use of this treatment is recommended for clinical use in situations of difficult retention and stability.
Yun, SD. et al., 2017 [101]	Beagle dog, M, 12 months	4	OSSH140 6 (MS) and OSSH1406HE(ES) (Osstem Implant, Seul, Republic of Korea), 6 mm × 1.4 mm; n = 56 (14 × 4) + 40 = 96	Machined and original AE, placed in the jaw of beagle dogs, removed at 4 weeks and treated to be reused and implanted in the jaw	n = 56; GA: jetair-water; GB: mechanical cleaning; GC: mechanical + chemical cleaning	n = 40 (20 MS + 20 ES); GD: unused, autoclave	8 and 12 s	SEM: differences on the surface; EDS: elemental composition; insertion torque tester; light microscopy: %BIC and %BV	Stability	Surface properties	Remaining compounds reduced in each cycle, without deformation. Biological response: %BIC and %BV higher at 4 s than at 8 s (*p* < 0.01), higher in ES than MS, but without significance; %BV of controls higher than experimental groups (*p* < 0.01), but without differences in %BIC.	Reused Ms can have a surface composition equivalent to new devices. The use of protocols with a mechanical and chemical component seemed to produce better results. The biological response already produces other variant results, which may imply that these treatments are not the most suitable from a biomechanical perspective.
Jang, T- H. et al., 2018 [102]	White New Zealand rabbit, M, 5 months	21 (two tibias)	Ti_6_Al_4_V (Osstem Implant, Seul, Republic of Korea), 6 mm × 1.4 mm, n = 126	n = 42 AE with nitric or hydrochloric acid. ECG: immediate immersion after EA in calcium chloride in a sealed location	n = 42, ECG: AE with calcium chloride immersion; n = 42, EG: AE without immersion	n = 42, CG no treatment	1, 4 and 7 s	Mechanical testing of cutting capacity and removal torque; SEM: analysis of changes in surface morphology	Stability	Capacity sharp	Surface: porous structures present in both experimental groups. RTVs: at 1 week were significantly higher in the experimental groups (*p* < 0.05), with no differences between EG and ECG; at 4 and 7 weeks, higher in the ECG group, and lower in the control group (*p* < 0.05); no changes in control over time.	We can increase the stability of Ms, increasing surface roughness by acid, and prevent contamination by calcium chloride.
Bakopoulor, A. et al., 2019 [103]	New Zealand rabbit, M, 4 months	6	The Sydney Mini Screw (Ti grade 5) + Aarhus (Aarhus system, American Orthodontics, Sheboygan, WI, USA), n = 24	iBGS; 8 weeks; injection through 1 cc cavity	T1: n = 8 SMS + IBGs; T2: n = 8 SMS	n = 8, no treatment and Aarhus	8 s	EA: stress point analysis; Micro-CT: stability; histological analysis	Stability primary	Dispersal from iBGs	All groups with cortical integration. iBGS group filled gaps, but with a greater inflammatory response, derived from a greater number of white blood cells (T0); at T8 complete healing—iBGS disperses with empty SMS. Control: uniform integration across the Ms in T0 and T8.	Biocompatibility and uniform integration with and without iBGS. Good delivery of iBGS to the bed, almost completely replacing the missing bone. Good results with the concentrations used.
Oga, Y. et al., 2019 [61]	Japanese white rabbit, F, 14 weeks	11	Dual-Top (Jeil Medical, Republic of Korea), 6 mm × 1.6 mm + auxiliary built-in device Ti_6_Al_4_V, F136-96(ASTM, West Conshohocken, PA, USA), PCT + Durometer silicone rings; n = 42	n = 22 Ti_6_Al_4_V auxiliary built-in device with two parts: capture of compressive forces + three peaks inserted into the bone cortex, X	n = 11 4 s; n = 11 8 s; Placing with auxiliary device	n = 9 4 s; n = 11 8 s; no treatment	4 and 8 s	Micro-CT: assessment of bone thickness, M/O interface and insertion depth	Stability	Bone thickness and insertion depth	Lateral displacement 0.01, 0.02 or 0.03 mm: device had significant effects on lateral displacement (*p* < 0.01), not taking time into account. Retention force: experimental at 4 s and 8 s greater than controls at all displacements.	The automatic anchoring auxiliary device coupled to the Ms increased its stability, on average, by 1.6 to 2.8×. It may be possible to allow the use of Ms that are smaller in length and diameter, fundamental characteristics in substrates that are more difficult to insert.
Yucesoy, T. et al., 2019 [104]	New Zealand rabbit, M, 9 months	18	Original SLA pure Ti, 8 mm × 1.8 mm, n = 72	Osseo Pulse LED, 618 nm, 20 mW/cm^2^, 5 min, for 21 days. Ozone therapy: (Ozonytron XL, Mymed); concentration 10–100 µg/mL, 90%, 30 s every 3 days	LED; Ozone. three sub-groups: immediate load G1, at 4 s G2 and at 8 s G3	Without treatment three sub-groups: immediate load G1, at 4 s G2 and at 8 s G3	0, 3, 4, 7, 8 and 11 s	SEM and IFM: surface characteristics biomechanical analysis. RR FA: stability assessment. %BIC analysis	Stability	Surface properties	Ozone G2 with significantly higher values of bone volume than the remaining G2, and the same was true for G3 photo and ozone. RFA: no statistical differences between groups. Histomorphometric analysis: ozone G2 and photo G2 with significantly higher % of osteointegrated areas than the G2 control, and in G3, photobiomodulation obtained greater values	Both photobiomodulation and ozone therapy are safe and effective methods for increasing bone volume, and therefore Ms stability, with implications not only in orthodontics, but in all areas that use implants.
Cho, Y.C. et al., 2021 [105]	White New Zealand rabbit,	12	Discs 316L 15 mm, 1.2 mm; n = 6 + Micro316L machined 2 mm × 10 mm, n = 6	AO:C_6_H_8_O_7_ and HF, 25 °C for 30 s + H cleaning. The anodizing (0.5, 1, 3.5 V) 5 min in H_2_SO_4_ solution 1 M at 98% and C_6_H_8_O_7_ 85% at 25 °C	n = 6 Anodizing	n = 6, no treatment	8 s	FE-SEM; XRD: crystallization phase; XPS: chemical analysis; histological analysis	Stability	Deposition of compounds; surface morphology	Group 5 V: deposition of double porous chromium oxide. Fibroblasts proliferate in all groups after 24 h, but in a well-distributed manner on anodized surfaces, adhering to flat and rough areas; anodized group shows larger osseointegration than I control.	Anodizing at 5 V on 316L BSS Ms can generate porous structures on their surface. This surface shows potential in improving cell adhesion and recovery in order to promote a higher survival rate of the Ms.
Choi, S- H. et al., 2021 [106]	Beagle dog, M, 12–15 months	8	Ti-6Al-4V (Orlus™, Ortholution, Seoul, Republic of Korea), 4/7 mm × 1.8 mm; n = 64 = 8 × 8	n = 32 UV light, 12 min, by TheraBeam Super Osseo device,	n = 32, UV light; G4mm and G7mm	n = 32, no treatment; G4mm and G7mm	7 and 28 days; 8 s	Measurement of success rate: mobility ≤ 1 mm (Periotest); Maximum insertion torque and micro-CT tests: %BA and %BV/TV analysis; histological analysis: %BIC	Stability	Success rate	Success rate: 100% in experimental 7 mm and 87.5% in control 7 mm. ITVs and RTVs: values increase with increasing length and surface treatment (*p* < 0.05). %BV/TV: trend equivalent to %BA, but without significant interaction. %BIC: values increase with the use of surface treatment and with length (*p* = 0.021 and *p* = 0.014, respectively).	Photofunctionalization using UV light can significantly increase biomechanical stability compared to untreated Ms.
Auciello, O. et al., 2022 [107]	Wistar rat M, X	10	Ti-6Al-4V AbsoAnchor (DENTOS Inc., Daegu, Republic of Korea); n = 20	Tungsten + UNCD 30 days; argon gas + argon gas/CH_4_ 0.8 sccm at 90 mbar + MO 1200 W	n = 10 Treatment UNCD	n = 10, no treatment	30 days	SEM and AFM; EDS: chemical composition; analysis with optical microscope: BIC	Tissue response Biocompatibility	Stability surface roughness	Larger W interface with greater roughness with UNCD ( + 0.3 nm rms); UNCD 3–5 nm film; UNCD granules increase surface roughness; osteointegration in both, but with control with more bone tissue. Average BIC% values: C—53.40 ± 13%; UNCD—58.82 ± 9%.	UNCD treatment with excellent biocompatibility. No differences in osteointegration. It can protect against titanium corrosion.
Im, C. et al., 2022 [30]	New Zealand White rabbit, M, 8 weeks	3	Ti–6Al–4V Ms ELI (Fort Wayne Metals Research Products Co., Fort Wayne, IN, USA) 3.3 mm × 1.4 mm	AO (HNO_3_ + HF+ H_2_O) 10 s + H_2_O + 1.4 wt% NH_4_F) 20 V, 60 min + P: 0.5 vol% silicate, 5 min + drying 1 h + 20× (0.05 M NaH_2_PO_4_ and Ca(OH)_2_, 90 °C, 1 min interval). Heating 500 °C, 2 h	AH: anodizing and heat treatment; APH: anodizing, pre-calcification and heat	UT: no treatment	4 s	Digital measurement of removal torque; FE-SEM: analysis of surface morphology; EDS: analysis of differences in elemental composition	Stability	Surface properties. Bioactivity. Cytotoxicity	Surface: AH—dense and aligned formation of nanotubes, with protrusions; APH—presence of granular Ca_3_(PO_4_)_2_ precipitate. Bioactivity: presence of Ca in the AH and APH groups, absence in the UT; FE-SEM: removal causes fractures not only at the interface (UT), but also in places of attached bone tissue, mainly in APH.	The APH surface obtained well-aligned nanotubes with a dense structure. Precipitates of calcium phosphate and hydroxyapatite were obtained in clusters, which allow greater binding to proteins that form new endogenous bone. Compared to the control, the experimental groups showed significantly higher removal torque.
Li, M. et al., 2022 [67]	Wistar rat, F, 8 months	144	Mini-pin (original SLA) 5.0 mm × 1.1 mm, n = 144	Immersion in simulated body fluid 24 h, 37 °C for BioCaP formation + immersion in SBF 5× 24 h, 37 °C for deposition of amorphous layer + 20 mL calcium phosphate solution	G2—No treatment, bath in PBS + BSA; G3 -amorphous treatment; G4—amorphous treatment + BSA; G5—TC; G6—TC+ BSA	G1: no treatment or BSA	3 days, 1, 2 and 4 s	SEM; FTIR; CLSM; spectrometry; activity n phosphatase alkaline and %BIC	Stability	Bioactivity by transport of biological agents	The thickness of crystalline layers is seven times greater than that of amorphous ones. Treatment by crystalline BioCaP allowed pharmacological transport. There was an increase in bone/Ms contact in the 1st week in G4, but in other groups this increase occurred at 2 and 4 weeks.	The pharmacological transport capacity was 10 times greater in the BioCaP treatment than in the amorphous ones. The contact between bone and Ms increased after the 1st week in the crystalline group, unlike the others, suggesting that this treatment is a technique that can increase stability and increase the success rate of Ms.
Seker, ED et al., 2022 [108]	White New Zealand rabbit, F, 12 months	14	Pure Ti grade IV, 8 mm × 1.8 mm, n = 56	n = 56, SLA: Al_2_O_3_ + AE hydrochloric and sulfuric, 90 °C, 15 min (G1) and 18 min (G2) + ultrasonic cleaning with acetone, 70% ethanol and H_2_O +sterilization by gamma rays	n = 28, G2: SLA of roughness 1.5 µm (no load and immediate load at 4 and at 8 weeks)	n = 28, G1: roughness SLA 1 µm (no load and immediate load at 4 and 8 weeks)	0, 4, 8 s	SEM, EDS, IFM, RFA, ISQ measurement and histological analysis	Stability	Morphology surface	Surface after use, without damage or deformation, and composition identical to mineralized bone. Stability: differences between initial ISQ values and differences between beginning and end without significance; there are intragroup differences (loading protocol), *p* < 0.05. BIC: in G2, higher values at 8 weeks and in immediate loading.	No significant differences in bone volume between groups. Greater stability in Ms subjected to loading after the healing period, instead of immediate loading: higher osseointegration rate in G2.
Byeon, S. et al., 2023 [31]	Sprague-Dawley rats, M, 8 weeks old	18	Ti-6Al-4V ELI alloy rods used to make Mis 3.3 mm × 1.4 mm, n = 36	APH: HNO_3_ + HF (H_2_O ratio of 12:7:81 for 10 s)+ ultrasonic cleaning in distilled water for 5 min + dryer at 50 °C for 24 h. SBF group: 10 mL of SBF for 5 days and 10 days, at 37 °C. Ibandronate groups: 10 min or 60 min in ibandronate ar −0.05 MpPa e and lyophilized with N_2_ gas + H_2_O for 7 days	2 Groups: n = 9 AN—immersion in SBF. N = 9 IB—immersion in ibandronate	n = 18 UN: untreated, machined	4 w	FE-SEM. RTV test	Stability	Surface properties	The removal torque values were higher in the AN and IB groups compared to the UN group, and the IB group showed the highest values. IB (8.48 ± 2.34 N/cm): statistically significant differences among all test groups. FE-SEM: UN—no adhesion to bone material and it showed the interfacial fracture; AN and IB—locally attached bone materials, with a mixture of cohesive and interfacial fractures, better observed in IB. All groups had new bone formation, but only IB had thick expansion of bone along the threads of Ms.	In the UN and AN groups, new bone was not formed or partially formed along the threads of the Ms. In the IB group, new bone was formed thickly along the threads. It was concluded that this method must be surface treatment to enhance the bioactivity and osseointegration, but it is necessary to study the biostability and biofunctionality on the surface of Ms according to the concentration of the drug using the sustained release method of the drug.
Nishioka-Sakamoto, K. et al., 2023 [69]	Wistar rats, M, 11 weeks old, 250–300 g	20	4.5 mm in length and 1.2 mm in diameter (ACE, Brockton, Mass)	Bone holes with different diameters (1.6, 2.1, or 2.5 mm) were drilled in the rat tibias. After a healing period of 2 or 4 weeks, orthodontic force was applied. The holes were filled with e β-TCP beta-tricalcium phosphate	n = 18 β-TCP	n = 20 untreated	0, 2 and 4 weeks	In vivo micro-CT, ex-vivo micro-CT, histological analysis, bone morphometry	Stability due to bone healing	Bone formation and percentage	The bone hole of 1.6 mm in diameter was employed as the OAS-loosening model. When b-TCP was inserted into the bone hole, the linear distance and mesial tipping angle of the OAS movement decreased markedly. The values of bone morphometry significantly increased with b-TCP filling.	An OAS-loosening model was established in rats and demonstrated that the loosening OAS was stabilized by b-TCP filling through bone formation. b-TCP may be useful for fixation of a loosening OAS.
Okawa, K. et al., 2023 [70]	Wistar rats, M, 25 weeks old, 400 g	24	Ti-6Al-4V titanium alloy, (Le Fort System, Pro-Seed, Japan) 3 mm × 1.2 mm, n = 48	Plasma surface treatment with a PZ2 piezo brush® (Relyon Plasma, Regensburg, Germany) for 30 s, with or without load	n = 24 plasma treated with loading (PTL) or without loading (PTU)	n = 24, no treatment	3, 5, 7 days and 2 weeks	Micro-CT: %BIC, bone density, histological analysis. Second harmonic generation imaging and XRD	Stability	Bone properties	Bone–implant contact rate increased more rapidly at an early stage in the treated surface group than in the untreated surface group. Collagen fiber bundle diameter showed that the measured values adjacent to regions A and D were significantly higher than those at regions B and C.	The results of bone contact rate and bone mineral content indicated that there was no significant difference between the surface-treated and untreated groups in the long term. Surface treatment of screws may accelerate initial bone remodeling.
Yamagata, K. et al., 2023 [71]	Japanese white rabbits, M, 3.5–4.0 kg	8	Dual-top (Jeil Medical, Republic of Korea), 5 mm × 1.3 mm. Kono Seisakusyo Corp, Chiba, Japan; raw material, Ti6Al4V, n = 32	Novel auxiliary device with spikes	n = 16, auxiliary device	n = 16, no treatment	28 and 56 days	Stability measurement Periotest. Histomorphometic analysis: BIC% and implantation spike depth	Stability	Implantation depth	The highest median PTV was 2.22 in the nonauxiliary group at 0 days and the lowest was 3.41 in the auxiliary group at 56 days. The interaction between the effects of the auxiliary device and days was not significant. There were no significant differences in BICs between the auxiliary and nonauxiliary groups at both 28 and 56 days	The results suggest that the use of Ms in conjunction with auxiliary devices provides stable skeletal anchorage, which may be useful in orthopedic treatments.
Lee, Y.T. et al., 2024 [109]	White New Zealand rabbit, M, 12 months	12	Ti (Mondeal Medical Systems, Germany) 9 mm × 2 mm, n = 48	Formation of nanotubes on the surface by AO in 1 M H_2_SO_4_ and NaF at 3 or 7 V for 5 h + heating at 300 °C for 3 h	Set 2, n = 6 Microporous group; sets 3–5, 3 n = 6 groups of nanopores on the right tibias. 7 V	Set 1, n = 24 Placebo control: non-porous on left tibias	12 s	Removal torque test with sensor; FE-SEM and SEM: composition surface and nanotubes	Stability	Surface morphology. Survival	Nanotube diameter increases with higher voltage and concentration; RTV: gradual increase from set1 to set 5, (*p* < 0.001). For 100% survival rate, minimum torque of 6.6 ± 0.8 N-cm and minimum thickness of 22.5 ± 4.8 nm.	Mts with nanoporous surfaces promoted higher survival rates and greater stability due to removal torques than microporous ones, the results being thought to be due to the greater thicknesses of TiO_2_ deposited on their surface by anodizing.

AAS: Atomic absorption spectrometry; AE: acid-etching AFM: atomic force microscopy; AO: oxidative anodizing; BA: bone area; BIC: bone interface contact; BSA: bovine serum albumin; BV/TV: ratio between bone volume and thickness volume; CLSM: confocal laser scanning microscope; DDL: distance between double labels; DMP-1: dentin matrix protein 1; EDX/EDS: X-ray spectrophotometry; FEA: finite element analysis; FE-SEM: field-emission scanning electron microscopy; FTIR: Fourier-transform infrared spectroscopy; FTV: fracture torque value; G: group; GCF: gingival crevicular fluid; HA—hydroxyapatite; IFM: infinite focus microscopy; iBGS: injectable bone graft substitute; ISQ: implant stability quotient; ITV: insertion torque value; LIPUS: low intensity pulsed ultrasound; LED: light emitting diode; LLLT: low-level laser therapy; MAR: mineral apposition rate; modSLA: modified SLA surface; MS/BS: mineralizing surface/bone surface ratio; MT—maximum torque; NPE—near peak energy; PICF: peri-implant crevicular fluid; PTV: Periotest value; UNCD: ultracrystalline diamond; RBM: resorbable blasting media; RFA: resonance frequency analysis; rhBMP-2: recombinant human bone morphogenetic protein 2; RTV: removal torque value; SEM: scanning electron microscopy; SL: sandblasting; SLA: sandblasting, large-grit and acid-etching; SL/NaOH: sandblasting and sodium hydroxide; Sr: strontium; TEV: total energy value; TIEV: total absorbed insertion energy value; TREV: total absorbed energy value of removal; UV: ultraviolet; vs.: versus; X: not clarified; XRD: X-ray diffractor. mineral apposition rate.

**Table 4:** Summary of extrapolated data from included clinical trials.

Author, Year	Study Design	Sex and Age	n	MI Type and Number	Surface Treatment	Experimental Group	Control Group	Period of Follow-Up	Test Used to Evaluate Outcomes	Primary Outcome	Secondary Outcomes	Results	Conclusions
Chaddad, K. et al., 2008 [16]	NRCT	13–65 years	10	DualTop™, Ti (C-implant, Seoul, Republic of Korea), n = 32 = 17 + 15	Source SLA surface	n = 15, C-implant (SLA surface)	n = 17, Dual-top (machined surface)	7, 14, 30, 60 and 150 days	Measurement of insertion torque	Success rate and stability	Surgical difficulty and post-surgical pain	SR: control 82.4%, SLA 93.4%. Devices always fail with torque < 15 Ncm. ITVs: influences SR (*p* < 0.05). Surface treatment did not create significant differences in SR.	The MI surface did not influence the SR of the same ITVs greater than 15 Ncm, which appear to be vital to the success of Ms under immediate loading.
Schaetzle, MA et al., 2009 [116]	RCT	21 M/19 F; 27.9 years	40	Orthosystem (Straumann, Basel, Switzerland) pure Ti (palatine), original SLA, 4.2 mm × 4.1 mm n = 40	modSLA (source SLA—0.25 grains- 0.5 mm and AE withHCl + H_2_SO4) + washing in N_2_ protection+ isotonic NaCl.	n = 20, modSLA	n = 20, SLA	0–84 days	RFA	Stability	-	All Ms with high insertion stability (average 39.25 N/cm). ISQ: Both groups showed a tendency to decrease, which reversed at 42 and 63 days, respectively, for the modSLA and control groups; at 12 weeks, modSLA greater than control.	The chemical modification of the SLA treatment positively influenced initial osseointegration through greater effectiveness of the healing process.
Calderon, J.H. et al., 2011 [113]	NRCT	6 M/7 F	13	IMTEK Ortho (3M, St. Paul, MN, USA), 6 mm mandible, 8 and 10 mm maxilla; n = 24	Source SLA	SLA of origin, maxillary group and mandibular group.	-	6 months	Overlay of occlusal radiographs and CBCT: displacement analysis	Stability	-	Angular displacement at 5 months: 65% ≤ 1°; 35% ≥ 2°. Less displacement in the mandibular group. Best results with 8 mm MI, without statistical differences. Length influences displacement variation.	Surface modification can improve osseointegration. All Ms were stable. No need for greater force to be applied when removing treated Ms.
Kim, SH. et al., 2012 [112]	Retro	1 M/7 F, 17–46 years	8	Source SLA (C-implant, Seoul, Republic of Korea), 8.5 mm × 1.8 mm, n = 16	Source SLA	SLA position after mass shrinkage	SLA starting position after placement (CBCT)	9 months	Overlay of CBCT DICOM data: 3D change analysis	Stability	-	Average RTV of 23.69 Ncm. Relative values of position changes of the tail and head on the *x*-axis (intrusion and extrusion) were −0.04 ± 0.19 mm and 0.01 ± 0.18 mm. No significant difference between before and after retraction.	The C Ms with SLA surface remained stable during the application of forces.
Noorollahian, S. et al., 2012 [39]	RCT	-	40	2 mm × 10 mm; n = 40 used on patients for 7 months	H_2_0 and drying +H_3_PO_4_ 37% 1 mL+ in NaOCl 5.25 mL (10 mL) for 30 min	n = 20 NP2: irrigation + drying; P2: irrigation + drying + phosphoric acid + NaOCl	n = 20, C2: autoclave	-	Torque measurements	Stability	-	ITVs: NP2 significantly smaller than P2 and C2, with no differences between them; RTVs and FTVs had no differences between all groups.	Absence of deleterious effects on the three torque values due to the use of AE, NaOCl and autoclave. Reused microimplants can be used.
Bratu, DC et al., 2014 [15]	NRCT	20–38 years	20	10 mm × 1.6 mm (MIS Implants Technologies, HaZafon, Israel), n = 40	SLA	n = 20, SLA	n = 20, untreated, machined	6 months	Measurement of insertion and removal torques	Stability	-	ITVs: control 18.55 ± 8.52 Ncm/SLA 20.45 ± 9.21 Ncm. RTVs: control 17.40 ± 8.18 Ncm/SLA 23.55 ± 9.68 Ncm.	SLA improved the secondary stability of Ms. 1.6 mm diameter suitable for good stability.
Ekizer, A. et al., 2016 [18]	RCT	7 M/13 F; 16.77 ± 1.41 years	20	Titanium, 8 mm × 1.6 mm, n = 40	LED: OsseoPulse LED (Biolux), 618 nm, 20 mW/cm^2^, 20 min/day, 21 days	n = 20, LED	n = 20, without treatment	0, 1, 7 days, 1, 2 and 3 months	RFA	Canine distalization rate	Stability + inflammation	ISQ: similar values between groups in the 1st month; different stability between groups in the 2nd and 3rd months (LED > control, *p* < 0.01).	LED can accelerate orthodontic tooth movement. Has a positive effect on stability No effects on IL-1β.
Flieger, R. et al., 2019 [115]	RCT	7 M/13 F; 32.5 ± 6.1 years	20	Grade V titanium, 10 mm × 1.4 mm; n = 40	LLLT 635 nm (Lasotronix SmartM), 100 mW, 10 J, 100 s per point, 2 times/session, 199.04 mW/cm^2^	n = 20 LLLT irradiation	n = 20, without treatment	0, 3, 6, 9, 12 15, 30 and 60 days	Periotest: stability analysis; NRS-11 Pain Rating Scale	Stability	Pain perception	Lower Periotest values in the experimental group indicate greater stability at 3, 30 and 60 days (*p* < 0.05 in both). No MI failed over the 60 days.	LLLT 635 nm promotes secondary Ms stability. There are, however, no effects on pain appreciation.
Park, H.J. et al., 2019 [117]	RCT	13 M/27 F; 22.16 years	40	1.6 mm × 6 mm; n = 98	AE	n = 49 AE	n = 49, without treatment, machined	-	Success rate. SEM. Torque measurement (Sensor Mark-10)	Stability + success rate	Mobility and topography	Topography of different roughness. SR: experimental group greater than control (91.8% vs. 85.7%), *p* = 0.323. Average ITVs: higher in the experimental group, but without differences in surface and jaw treatment.	Higher SR in the experimental group, without statistically significant difference. The same occurred in primary stability.
Kim, SH. et al., 2012 [112]	Retro	1 M/7 F, 17–46 years	8	Source SLA (C-implant, Seoul, Republic of Korea), 8.5 mm × 1.8 mm, n = 16	Source SLA	SLA position after mass shrinkage	SLA starting position after placement (CBCT)	9 months	Overlay of CBCT DICOM data: 3D change analysis	Stability	-	Average RTV of 23.69 Ncm. Relative values of position changes of the tail and head on the *x*-axis (intrusion and extrusion) were −0.04 ± 0.19 mm and 0.01 ± 0.18 mm. No significant difference between before and after retraction.	The C Ms with SLA surface remained stable during the application of forces.
Matys, J. et al., 2020 [114]	RCT	8 M/14 F; 31.7 ± 9.7 years	22	Grade V titanium, 10 mm × 1.4 mm; n = 44	LLLT 808 nm (SmartM Pro by Lasotronix), 100 mW, 4 J, 40 s/point, 2 times/session, 200 mW/cm^2^	n = 22 LLLT irradiation	n = 22, without treatment (loss of 1)	0, 3, 6, 9, 12 15, 30 and 60 days	Periotest: stability analysis; NRS-11 Pain Rating Scale	Stability	Pain perception	Stability: PTVs with significantly higher values in controls than in irradiated groups (*p* = 0.009). At 30 days, controls had a greater decrease in stability (*p* = 0.004).	LLLT increased secondary stability after 1 and 2 months of implantation. There were no differences in pain scores.
Rampurawala, A. et al., 2020 [14]	RCT	18–45 years	17	AbsoAnchor (DENTOS Inc., Daegu, Republic of Korea), Teeth; 7 mm × 1.4 mm; n = 34 (24 after deletion)	UV-A rays (15 W, 350 ± 20 nm, 0.1 mW/cm^2^) and UV-C (15 W, 250 ± 20 nm, 2.0 mW/cm^2^), 15 min (before insertion)	n = 12, UV light	n = 12, no treatment	6–8 months	SEM: contact O/M; EDX: Deposition of elements on the surface	Stability	Hydrophilicity and deposition of calcium and phosphorus	Increased hydrophilicity of the titanium surface. O/M Contact: higher score in the experimental group, with no statistical difference. Calcium and phosphorus deposition: no significant differences between groups.	UV light converted Ms surface: hydrophilic to superhydrophilic. Contact with the bone was greater in the lower region of the Ms in both groups.
Moghaddam, S. et al., 2021 [21]	RCT	8 M/23; 18.5 years	31	Dual-Top Anchor System (Jeil Medical, Republic of Korea),10 mm × 2 mm, n = 62	SLA: alumina particles 250 µm, 4 MPa + ultrasonic cleaning with acetone, 75% ethanol and H_2_O, 15 min + immersion in 0.11 HF mol/L and 0.09 mol/L HNO_3_, 25 °C, 10 min +drying at 50 °C, 24 h	n = 31, SLA	n = 31, no treatment	3, 6, 10, 14, 18 weeks	Measurement clinical performance and mobility (<1 mm). Digital measurement of insertion and removal torques	Success rate	Stability	SR: SLA 90.32% vs. 83.87% control (*p* > 0.05); Success rate lower the lower the age in both groups (*p* = 0.025): <15 years 66.66% and >15 years 95.45%. Mean RTVs (N.cm) higher in the SLA group (*p* < 0.05): SLA 15.71 ± 5.563 and C 8.08 ± 2.481. No significant differences in mean ITVs (N.cm): 12.10 ± 6.295 vs. C 12.42 ± 5.755.	Surface roughness of orthodontic IMs by SLA treatment had no influence on the success rate, but increased removal torques significantly.
Manni, A. et al., 2022 [17]	RCT	23 F/16 M; 15.55 ± 7.91	39	Machined and original AE (Osstem Implant, Seul, Republic of Korea), 1.2–1.4 mm × 8 mm, n = 78	AE of origin	n = 39, AE of origin	n = 39, untreated, machined	9.3 months ± 1.31	Measuring success rate: clinical performance analysis	Success rate	Stability	Failure rate: 25.6% control and 28.2% experimental. Cumulative SR of 26.9%. Average survival of failures: 52 days ± 58. No influence of surface treatment, Ms location, gender and diameter on stability.	The success rate did not vary statistically significantly between groups in cases of anchorage reinforcement during treatment with the Herbst device.
Durrani, O. et al., 2023 [110]	RCT	32 M/60 F, 18 ± 1.8 years	92	Ti-6Al-4V alloy, 8 mm × 1.6 mm, self-drilling and self-tapping, n = 184	Precipitation of HA: ultrassonic acetone cleaning 10 min + H_2_O + 20 mL CaCl_2_ 0.1 mol/L + 20 mL K_2_HPO_4_ solution 0.1 mol/L with stirring for 60 min + H_2_O cleaning + autoclaving	n = 92, HA (46 on first quadrant, 46 on left quadrant)	n = 92, no treatment (46 on first quadrant, 46 on left quadrant)	Monthly over 18 months	Number of failing miniscrews	Failure rate	Stability	Non-significant statistical difference. Better survivability of the HA-coated Ms from the second to the fifth month, but *p* = 0.67. Left side failed more (19.6%vs4.3%, *p* = 0.002).	Ms coated with HA do not have any statistical difference in the failure when placed on the buccal shelf of the maxilla. The same method of using biomimetic precipitation of HA incorporated with other drugs on surfaces of Ms.
Ravi, J. et al., 2023 [111]	RCT	9 F, 23–29 years	9	Self-drilling, tapered Ti Ms of 8-mm × 2 mm (A1 orthodontic Ms (Bioray Enterprises, Taipei, Taiwan)	SLA: sandblasted with large-grit alumina, followed by acid-etching with hydrochloric acid and sulfuric acid	n = 18, SLA	n = 18, no treatment	4 weeks	Torque test with digital torque meter TQ-8800	Stability	Failure rate	The mean maximum insertion torque was 17.9 ± 5.6 Ncm for surface-treated Ms and 16.4 ± 9.0 Ncm for non-surface-treated Ms. The mean maximum removal torque was 8.1 ± 2.9 Ncm for surface-treated Ms and 3.3 ± 1.9 Ncm for non-surface-treated. Among the failed Ms, 71.4% were non-surface-treated Ms and 28.6% were surface-treated Ms.	The insertion torque and failure rate did not differ significantly between the groups, whereas the removal torque was significantly higher in the surface-treated group. Thus, surface treatment using sandblasting and acid-etching may improve the secondary stability of self-drilling Ms.

AE: acid-etching; BA: bone area; BIC: bone interface contact; BV/TV: ratio between bone volume and thickness volume; C2: control group 2; EDX/EDS: X-ray spectrophotometry; FE-SEM: field-emission scanning electron microscopy; F—female; FTV: fracture torque value; HA: hydroxyapatite; HCL: hydrochloric acid; H_2_0: water; H_2_SO_4_: sulfuric acid; ISQ: implant stability quotient; ITV: insertion torque value; LED: light-emitting diode; LLLT: low-level laser therapy; M: male; MD: mesiodistal; MI—microimplant; min—minutes; n: sample size; NaCl: sodium chloride; NaOCl: sodium hypochlorite; NP2: wash group 2; NRCT: non-randomized controlled trial; PTV: Periotest value; RCT: randomized controlled trial; Q2: acid-etching group 2; Retro: retrospective study; RFA: resonance frequency analysis; RTV: removal torque value; SEM: scanning electron microscopy; SLA: sandblasting, large-grit and acid-etching; s: seconds; UV: ultraviolet; SR: success rate; X: not clarified.

## Data Availability

The data presented in this study are available on request from the corresponding author.

## Supplementary Materials

The following supporting information can be downloaded at: https://www.mdpi.com/article/10.3390/jfb15030068/s1, Table S1: Search Strategies/Phrases; Table S2: Assessment of Risk of Bias for Randomized Controlled Clinical Trials; Table S3: Assessment of Risk of Bias for Non-Randomized Clinical Trials; Table S4: Assessment of Risk of Bias for In vivo Studies; Table S5: Assessment of Risk of Bias for in vitro studies.
