# Supplementary material for: Surface Treatment of Dental Mini-Sized Implants and Screws: A Systematic Review with Meta-Analysis
Source: J Funct Biomater. 2024 Mar 10;15(3):68. doi: 10.3390/jfb15030068 (PMC10971647; doi:10.3390/jfb15030068)
Supplement: Supplementary file 1 [file jfb-15-00068-s001.zip › jfb-2863108-supplementary.pdf]

Supplementary Materials

Surface Treatment of Dental Mini-Sized Implants and Screws: A Systematic Review with Meta-Analysis

Ana Luísa Figueiredo [1], Raquel Travassos [1,2,3,4], Catarina Nunes [1,2,3,4], Madalena Prata Ribeiro [1], Mariana Santos [1], Flavia Iaculli [5], Anabela Baptista Paula [1,2,3,4,6,*], Carlos Miguel Marto [2,3,4,6,7], Francisco Caramelo [2,3,4], Inês Francisco [1,2,3,4,*,†] and Francisco Vale [1,2,3,4,†]

[1] Institute of Orthodontics, Faculty of Medicine, University of Coimbra, 3000-354 Coimbra, Portugal; uc2018277869@student.uc.pt (A.L.F.); uc47231@uc.pt (R.T.); uc47230@uc.pt (C.N.); uc2020181154@student.uc.pt (M.P.R.); uc2014107642@student.uc.pt (M.S.); fvale@fmed.uc.pt (F.V.)

[2] Laboratory for Evidence-Based Sciences and Precision Dentistry (LACBE–MDP), Faculty of Medicine, University of Coimbra, 3000-354 Coimbra, Portugal; cmiguel.marto@uc.pt (C.M.M.); fcaramelo@fmed.uc.pt (F.C.)

[3] Institute for Clinical and Biomedical Research (iCBR), Area of Environment Genetics and Oncobiology (CIMAGO), Faculty of Medicine, University of Coimbra, 3000-354 Coimbra, Portugal

[4] Clinical Academic Center of Coimbra (CACC), 3000-354 Coimbra, Portugal

[5] Department of Neurosciences, Reproductive and Odontostomatological Sciences, University of Naples "Federico II", 80138 Naples, Italy; flavia.iaculli@unina.it

[6] Institute of Integrated Clinical Practice, Faculty of Medicine, University of Coimbra, 3000-354 Coimbra, Portugal

[7] Institute of Experimental Pathology, Faculty of Medicine, University of Coimbra, 3000-354 Coimbra, Portugal

* Correspondence: anabelabppaula@sapo.pt (A.B.P.); ifrancisco@fmed.uc.pt (I.F.)

† These authors contributed equally to this work.

Table S1. Search Strategies/Phrases.

Database	ID	Search Strategy
**PubMed *via* Medline**		(Miniscrew* OR mini-screw* OR mini-implant* OR “mini implant*” OR “Mini Dental Implant*” OR miniscrew* OR micro-implant* OR “Dental Implants, Mini” OR “Dental Implant, Mini” OR “Skeletal anchorage” OR “absolute anchorage” OR “Temporary anchorage device*” OR "Orthodontic Anchorage Procedures"[Mesh] OR “Orthodontic Anchorage Procedure*” OR “Anchorage Procedure, Orthodontic” OR “Anchorage Procedures, Orthodontic” OR “Procedure, Orthodontic Anchorage” OR “Procedures, Orthodontic Anchorage” OR “Orthodontic Anchorage Technique*” OR “Anchorage Technique, Orthodontic” OR “Anchorage Techniques, Orthodontic” OR “Technique, Orthodontic Anchorage” OR “Techniques, Orthodontic Anchorage”) AND ("Surface Properties"[Mesh] OR surface* OR "Osseointegration"[Mesh] OR Osseointegration OR “Endosseous Healing” OR “Healing, Peri-implant Endosseous” OR “Healings, Peri-implant Endosseous” OR “Mechanical stability”) AND ("Orthodontics"[Mesh] OR Orthodontic*)
**Cochrane Library**	ID	
	#1	Search
	#2	Miniscrew*
	#3	mini-screw*
	#4	mini-implant*
	#5	"mini implant*"
	#6	“Mini Dental Implant*”
	#7	miniscrew*
	#8	micro-implant*
	#9	“Dental Implants, Mini”
	#10	“Dental Implant, Mini”
	#11	“Skeletal anchorage”
	#12	“absolute anchorage”
	#13	“Temporary anchorage device*”
		MeSH descriptor: [Orthodontic Anchorage Procedures] explode all trees
	#14	“Orthodontic Anchorage Procedure*”
	#15	“Anchorage Procedure, Orthodontic”
	#16	“Anchorage Procedures, Orthodontic”
	#17	“Procedure, Orthodontic Anchorage”
	#18	“Procedures, Orthodontic Anchorage”
	#19	“Orthodontic Anchorage Technique*”
	#20	“Anchorage Technique, Orthodontic”
	#21	“Anchorage Techniques, Orthodontic”
	#22	“Technique, Orthodontic Anchorage”
	#23	“Techniques, Orthodontic Anchorage”
	#24	MeSH descriptor: [Surface Properties] explode all trees
		surface*
	#25	MeSH descriptor: [Osseointegration] explode all trees
	#26	Osseointegration
		“Endosseous Healing”
	#27	“Healing, Peri-implant Endosseous”
	#28	“Healings, Peri-implant Endosseous”
	#29	“Mechanical stability”
	#30	MeSH descriptor: [Orthodontics] explode all trees
	#31	Orthodontic*
	#32	(#1 OR #2 OR #3 OR #4 OR #5 OR #6 OR #7 OR #8 OR #9 OR #10 OR #11 OR #12 #13 OR #14 OR #15 OR #16 OR #17 OR #18 OR #19 OR #20 OR
	#33	#21 OR #22 OR #23) AND (#24 OR #25 OR #26 OR #27 OR #28 OR #29 OR #30 OR #31) AND (#32 OR #33)
	#34	
**Embase**		(miniscrew*:ti,ab,kw OR 'mini screw*':ti,ab,kw OR 'mini implant*':ti,ab,kw OR 'mini dental implant*':ti,ab,kw OR miniscrew*:ti,ab,kw OR 'micro implant*':ti,ab,kw OR 'dental implants, mini':ti,ab,kw OR 'dental implant, mini':ti,ab,kw OR 'skeletal anchorage':ti,ab,kw OR 'absolute anchorage':ti,ab,kw OR 'temporary anchorage device*':ti,ab,kw OR tad:ti,ab,kw OR tads:ti,ab,kw OR 'orthodontic anchorage'/exp OR 'orthodontic anchorage procedure*':ti,ab,kw OR 'anchorage procedure, orthodontic':ti,ab,kw OR 'anchorage procedures, orthodontic':ti,ab,kw OR 'procedure, orthodontic anchorage':ti,ab,kw OR 'procedures, orthodontic anchorage':ti,ab,kw OR 'orthodontic anchorage technique*':ti,ab,kw OR 'anchorage technique, orthodontic':ti,ab,kw OR 'anchorage techniques, orthodontic':ti,ab,kw OR 'technique, orthodontic anchorage':ti,ab,kw OR 'techniques, orthodontic anchorage':ti,ab,kw) AND ('surface property'/exp OR surface*:ti,ab,kw OR 'osseointegration'/exp OR osseointegration:ti,ab,kw OR 'endosseous healing':ti,ab,kw OR 'healing, peri-implant endosseous':ti,ab,kw OR 'healings, peri-implant endosseous':ti,ab,kw OR 'mechanical stability':ti,ab,kw) AND ('orthodontics'/exp OR orthodontic*:ti,ab,kw
**Web of Science**		(Miniscrew* OR mini-screw* OR mini-implant* OR “mini implant*” OR “Mini Dental Implant*” OR miniscrew* OR micro-implant* OR “Dental Implants, Mini” OR “Dental Implant, Mini” OR “Skeletal anchorage” OR “absolute anchorage” OR “Temporary anchorage device*” OR “Orthodontic Anchorage Procedure*” OR “Anchorage Procedure, Orthodontic” OR “Anchorage Procedures, Orthodontic” OR “Procedure, Orthodontic Anchorage” OR “Procedures, Orthodontic Anchorage” OR “Orthodontic Anchorage Technique*” OR “Anchorage Technique, Orthodontic” OR “Anchorage Techniques, Orthodontic” OR “Technique, Orthodontic Anchorage” OR “Techniques, Orthodontic Anchorage”) AND (surface* OR Osseointegration OR “Endosseous Healing” OR “Healing, Peri-implant Endosseous” OR “Healings, Peri-implant Endosseous” OR “Mechanical stability”) AND (Orthodontic*)

Table S2. Assessment of Risk of Bias for Randomized Controlled Clinical Trials.

	Randomization Process	Deviations from the Intended	Missing Outcome Data	Measurement of Outcome	Selection of the Reported Result	Overall Bias
Noorollahian, S. et al. 2012 [39]	Y	Y	Y	Y	Y	Y
Park, H.J. et al. 2019 [117]	Y	Y	Y	Y	Y	Y
Rampurawala, A. et al. 2020 [14]	Y	Y	Y	Y	Y	Y
Matys, J. et al. 2020 [114]	Y	Y	Y	SC	Y	SC
Flieger, R. et al. 2019 [115]	Y	Y	Y	SC	Y	SC
Ekizer, A. et al. 2016 [18]	Y	Y	Y	Y	Y	Y
Schaetzle, M.A. et al. 2009 [116]	Y	Y	Y	Y	Y	Y
Manni, A. et al. 2022 [17]	Y	Y	Y	Y	Y	Y
Moghaddam, S. et al. 2021 [21]	N	Y	Y	Y	Y	N
Durrani, O. et al, 2023[110]	Y	SC	Y	Y	Y	SC
Ravi, J. et al, 2023[111]	Y	Y	Y	Y	Y	Y

Y – yes; SC – some concerns, N - no.

Table S3. Assessment of Risk of Bias for Non-Randomized Clinical Trials.

	Bias Due to Confounding	Selection of Participants	Classification of Interventions	Desviations from Intended Interventions	Missing Data	Measurement of Outcomes	Selection of the reported Result	Overall Bias
Calderón, J.H. et al. 2019[113]	Y	Y	Y	Y	Y	M	Y	M
Kim, S-H. et al. 2012[112]	Y	Y	Y	Y	Y	M	Y	M
Bratu, D. C. et al. 2014[15]	Y	Y	Y	Y	Y	M	Y	M
Chaddad, K. et al. 2008[16]	Y	Y	Y	Y	Y	M	Y	M
Kim, S-H. et al. 2008[73]	Y	Y	Y	Y	Y	M	Y	M

Y - yes, M - moderate.

Table S4. Assessment of Risk of Bias for In vivo Studies.

	Sequence Generation	Baseline Charcteristics	Allocation Concealment	Random Housing	Blinding	Random Outcome Assessment	Blinding of outcome evaluation	Incomplete Outcome Data	Selective Outcome reporting	Other sources of bias
Auciello, O. et al. 2022[107]	N	Y	N	U	N	Y	Y	Y	Y	Y
Bakopoulou, A. et al. 2019[103]	Y	Y	Y	Y	U	Y	U	Y	Y	Y
Miura, K. et al. 2014[86]	N	Y	N	U	N	Y	Y	Y	Y	Y
Cho, Y-C. et al. 2021[105]	Y	Y	N	Y	N	Y	U	Y	Y	Y
Niwa, K. et al. 2009[76]	N	Y	N	U	N	Y	U	U	Y	Y
Cho, Y-C. et al. 2013[83]	N	Y	N	N	N	Y	U	Y	Y	Y
Yadav, S. et al. 2015[50]	Y	Y	Y	Y	Y	Y	Y	Y	Y	Y
Kim, H-Y. et al. 2016[52]	N	Y	N	U	N	Y	N	Y	Y	Y
Lee, Y-T. et al. 2024[109]	Y	Y	N	Y	N	Y	N	Y	Y	Y
Seker, E.D. et al. 2022[108]	Y	Y	N	U	N	Y	N	Y	Y	Y
Li, M. et al. 2022[67]	N	Y	N	Y	N	Y	Y	Y	Y	Y
Miyawaki, S. et al. 2015[45]	N	Y	N	N	N	U	N	Y	Y	N
El-Wassefy, N. et al. 2015[88]	N	Y	N	U	N	Y	N	Y	Y	Y
Jang, I. et al. 2017[99]	N	Y	N	Y	N	Y	N	N	Y	Y
Karmarker, S. et al. 2012[80]	N	Y	N	Y	N	Y	N	Y	Y	Y
Jang, I. et al. 2015[90]	N	Y	N	Y	N	Y	U	Y	Y	Y
Tabuchi, M. et al. 2015[49]	N	Y	N	Y	N	Y	N	Y	Y	Y
Jang, T-H. et al. 2018[102]	Y	Y	N	Y	N	Y	U	N	Y	Y
Kim, T-W. et al. 2008[74]	N	Y	N	U	N	N	N	Y	N	Y
Yun, S-D. et al. 2017[101]	N	Y	N	Y	N	U	N	N	Y	Y
Oh, E-J. et al. 2014[44]	N	Y	N	Y	N	Y	Y	U	Y	Y
Takahashi, M. et al. 2016[98]	N	Y	N	N	N	Y	Y	Y	Y	Y
Oga, Y. et al. 2019[61]	N	Y	N	U	N	Y	Y	U	Y	Y
Oh, N-H. et al. 2014[87]	Y	Y	N	Y	N	Y	Y	Y	Y	Y
Choi, S-H. et al. 2021[106]	Y	Y	N	Y	N	Y	Y	Y	Y	Y
Bayani, S. et al. 2016[94]	N	Y	N	Y	N	Y	U	Y	Y	Y
Choi, S-H. et al. 2016[96]	Y	Y	Y	Y	N	Y	Y	Y	Y	Y
Yucesoy, T. et al. 2019[104]	Y	Y	N	Y	N	Y	N	Y	Y	Y
Sirisa-Ard, A. et al. 2015[91]	N	Y	N	Y	N	Y	U	Y	Y	Y
Goymen, M. et al. 2015[89]	Y	Y	U	U	N	Y	N	Y	Y	Y
Im, C. et al. 2022[30]	N	Y	N	Y	N	Y	N	Y	Y	Y
Cho, I.C. et al. 2012[78]	Y	Y	N	Y	N	Y	Y	Y	Y	Y
Fernandes, D.J. et al. 2017[20]	N	Y	N	Y	N	Y	U	Y	Y	Y
Pinto, M. et al. 2013[84]	Y	Y	N	Y	N	Y	Y	Y	Y	Y
Kang, H.K. et al. 2016[51]	Y	Y	N	Y	N	Y	U	Y	Y	Y
Cuairán, C. et al. 2014[85]	Y	Y	N	Y	N	Y	Y	Y	Y	Y
Ganzorig, K. et al. 2015[47]	N	Y	N	Y	N	U	N	Y	Y	Y
Omasa, S. et al. 2012[81]	N	Y	N	Y	N	Y	Y	Y	Y	Y
Gansukh, O. et al. 2016[97]	N	Y	N	Y	N	Y	Y	Y	Y	Y
Cha, B-K. et al. 2016[95]	N	Y	N	Y	N	Y	Y	Y	Y	Y
Mo, S.S. et al. 2010[77]	Y	Y	N	Y	U	Y	Y	Y	Y	Y
Uysal, T. et al. 2012[82]	Y	Y	N	Y	U	Y	Y	Y	Y	U
Kim, S-H. et al. 2009[13]	Y	Y	U	Y	U	Y	Y	Y	Y	Y
Espinar-Escalona, E. et al. 2016[28]	N	Y	N	Y	N	Y	N	Y	Y	Y
Vilani, G. et al. 2015[93]	N	Y	N	Y	N	Y	N	Y	Y	Y
Liang, Y. et al. 2015[48]	Y	Y	U	Y	U	Y	U	Y	Y	Y
Choi, S-H. et al. 2012[79]	N	Y	N	N	N	N	N	Y	Y	U
Chang, C-S. et al. 2009[75]	N	Y	N	N	N	U	N	Y	N	Y

Maino, B. et al. 2017[100]	Y	Y	N	Y	U	Y	N	Y	Y	Y
Aoki, T, et al. 2005[72]	N	Y	N	U	N	Y	N	Y	Y	Y
Tabuchi, M. et al. 2015[92]	N	Y	N	U	N	Y	Y	Y	Y	Y
Hassan, A. et al. 2003[68]	N	Y	N	Y	N	Y	N	Y	Y	Y
Byeon, S. et al. 2023[31]	N	Y	N	U	N	U	U	Y	U	Y
Nishioka-Sakamoto, K. et al 2023[69]	Y	Y	U	Y	Y	Y	U	Y	Y	Y
Okawa, K. et al 2023[70]	Y	Y	Y	Y	U	Y	Y	Y	Y	Y
Yamagata, K. et al, 2023[71]	Y	Y	U	U	U	U	U	Y	Y	Y

Y- yes, U – unclear, N – No.

Table S5. Assessment of Risk of Bias for in vitro studies.

	Sturctured summary	Scientific Background and Explanation of Rationale	Specific Objectives and/or Hypotheses	Intervention for Each Group	Outcome	Sample size	Random Allocation	Allocation Concealment Mechanism	Implementation	Blinding	Statistical Methods	Outcomes and Estimation	Limitations	Funding	Protocol
Noorollahian, S. et al. 2012[39]	Y	Y	Y	Y	Y	N	Y	N	Y	Y	Y	Y	N	Y	N
Fleischmann, L. et al. 2015[46]	Y	Y	Y	Y	Y	N	N	N	N	N	Y	Y	Y	Y	N
Ly, N. et al. 2019[60]	Y	Y	Y	Y	Y	N	N	N	N	N	Y	Y	N	Y	N
Pop, S. et al. 2017[53]	Y	Y	Y	Y	Y	N	Y	N	N	N	Y	Y	Y	Y	N
Iodice, G. et al. 2019[58]	Y	Y	Y	Y	Y	N	N	N	N	N	Y	Y	Y	Y	N
Yadav, S. et al. 2015[50]	Y	Y	Y	Y	Y	N	N	N	N	N	Y	Y	N	Y	N
Kim, H-Y. et al. 2016[52]	Y	Y	Y	Y	Y	N	Y	N	N	N	Y	Y	Y	Y	N
Li, M. et al. 2022[67]	Y	Y	Y	Y	Y	N	N	N	N	N	Y	Y	Y	Y	N
Miyawaki, S. et al. 2015[45]	Y	Y	Y	Y	Y	N	N	N	N	N	Y	Y	Y	Y	N
Iwanami-Kadowaki, K. et al. 2021[65]	Y	Y	Y	Y	Y	N	N	N	N	N	Y	Y	Y	Y	N
Pavlic, A. et al. 2019[62]	Y	Y	Y	Y	Y	Y	N	N	N	N	Y	Y	Y	Y	N
Zogheib, T. et al. 2021[66]	Y	Y	Y	Y	Y	N	N	N	N	N	Y	Y	N	Y	N
Mattos, C. T. et al. 2011[35]	Y	Y	Y	Y	Y	N	Y	N	N	N	Y	Y	Y	N	N
Estelita, S. et al. 2014[43]	Y	Y	Y	Y	Y	N	Y	N	N	N	Y	Y	Y	N	N
Alavi, S. et al. 2020[63]	Y	Y	Y	Y	Y	N	Y	Y	Y	Y	Y	Y	Y	Y	N
Galli, C. et al. 2012[38]	Y	Y	Y	Y	Y	N	N	N	Y	Y	Y	Y	Y	Y	N
Tabuchi, M. et al. 2015[49]	Y	Y	Y	Y	Y	N	N	N	N	N	Y	Y	Y	N	N
Tozlu, M. et al. 2013[42]	Y	Y	Y	Y	Y	N	N	N	N	N	Y	Y	Y	N	N
Akyalcin, S. et al. 2013[40]	Y	Y	Y	Y	Y	Y	Y	Y	Y	Y	Y	Y	Y	N	N
Muguruma, T. et al. 2011[36]	Y	Y	Y	Y	Y	N	N	N	N	N	Y	Y	Y	N	N
Tejani, H. et al. 2017[54]	Y	Y	Y	Y	Y	N	Y	N	N	N	Y	Y	N	N	N
Oh, E-J. et al. 2014[44]	Y	Y	Y	Y	Y	N	Y	N	N	N	Y	Y	N	Y	N
Giri, M. et al. 2020[64]	Y	Y	Y	Y	Y	Y	N	N	N	N	Y	Y	Y	Y	N
Oga, Y. et al. 2019[61]	Y	Y	Y	Y	Y	N	N	N	N	N	Y	Y	Y	Y	N
Im, C. et al. 2022[30]	Y	Y	Y	Y	Y	N	N	N	N	N	Y	Y	N	Y	N
Cho, I.S. et al. 2012[37]	Y	Y	Y	Y	Y	N	N	N	N	N	Y	Y	Y	N	N
Hergel, C. et al. 2019[57]	Y	Y	Y	Y	Y	N	Y	Y	N	N	Y	Y	Y	Y	N
Fernandes, D.J. et al. 2017[20]	Y	Y	Y	Y	Y	N	N	N	N	N	Y	Y	N	Y	N
Mattos, C.T. et al. 2010[29]	Y	Y	Y	Y	Y	N	N	N	N	N	Y	Y	N	Y	N
Kang, H.K. et al. 2016[51]	Y	Y	Y	Y	Y	N	Y	N	N	N	Y	Y	Y	N	N
Ganzorig, K. et al. 2015[47]	Y	Y	Y	Y	Y	N	N	N	N	N	Y	Y	Y	Y	Y
Kaci, N. et al. 2018[55]	Y	Y	Y	Y	Y	N	N	N	N	N	N	Y	N	N	N
Serra, G. et al. 2013[41]	Y	Y	Y	Y	Y	N	N	N	N	N	Y	Y	Y	N	N
Jongwannasiri, C. et al. 2019[59]	Y	Y	Y	Y	Y	N	N	N	N	N	N	Y	N	Y	N
Espinar-Escalona, E. et al. 2016[28]	Y	Y	Y	Y	Y	N	N	N	N	N	Y	Y	N	Y	N
Liang, Y. et al. 2015[48]	Y	Y	Y	Y	Y	N	N	N	N	N	Y	Y	N	Y	N
Pop, S. et al. 2018[56]	Y	Y	Y	Y	Y	N	N	N	N	N	Y	Y	N	Y	N
Baser, B. et al. 2023[34]	Y	Y	Y	Y	Y	N	N	N	N	Y	Y	Y	Y	Y	N
Byeon, S. et al. 2023[31]	Y	Y	Y	Y	Y	N	N	N	N	N	Y	Y	Y	Y	N
Gezer, P. et al. 2023[32]	Y	Y	Y	Y	Y	Y	N	N	N	N	Y	Y	Y	Y	N
Li, M. et al 2023[33]	Y	Y	Y	Y	Y	N	N	Y	N	Y	Y	Y	Y	Y	N

Y– yes, N - no.
